# Poly(Ionic Liquid) Electrolytes at an Extreme Salt Concentration for Solid-State Batteries

**DOI:** 10.1021/jacs.4c12616

**Published:** 2024-11-19

**Authors:** Shinji Kondou, Mohanad Abdullah, Ivan Popov, Murillo L. Martins, Luke A. O’Dell, Hiroyuki Ueda, Faezeh Makhlooghiazad, Azusa Nakanishi, Taku Sudoh, Kazuhide Ueno, Masayoshi Watanabe, Patrick Howlett, Heng Zhang, Michel Armand, Alexei P. Sokolov, Maria Forsyth, Fangfang Chen

**Affiliations:** 1 Institute for Frontier Materials, Deakin University, Burwood, VIC 3125, Australia; 2 ARC Industry Transformation Training Centre for Future Energy Technologies, Deakin University, Burwood, VIC 3125, Australia; 3 Department of Materials Engineering Science, Osaka University, 1-3, Machikaneyama, Toyonaka, Osaka 560-8531, Japan; 4 Department of Chemistry and Life Science, Yokohama National University, 79-5 Tokiwadai, Hodogaya-ku, Yokohama 240-8501, Japan; 5 Department of Chemistry, University of Tennessee, Knoxville, Tennessee 37996, United States; 6 University of Tennessee - Oak Ridge Innovation Institute, University of Tennessee, Knoxville, Tennessee 37996, United States; 7 Advanced Chemical Energy Research Centre (ACERC), Institute of Advanced Sciences, Yokohama National University, 79-5 Tokiwadai, Hodogaya-ku, Yokohama 240-8501, Japan; 8 Key Laboratory of Material Chemistry for Energy Conversion and Storage (Ministry of Education), School of Chemistry and Chemical Engineering, Huazhong University of Science and Technology, Luoyu Road 1037, Wuhan 430074, China; 9 Center for Cooperative Research on Alternative Energies (CIC energiGUNE), Basque Research and Technology Alliance (BRTA), Vitoria Gasteiz 01510, Spain; 10 Chemical Sciences Division, Oak Ridge National Laboratory, Oak Ridge, Tennessee 37831, United States

## Abstract

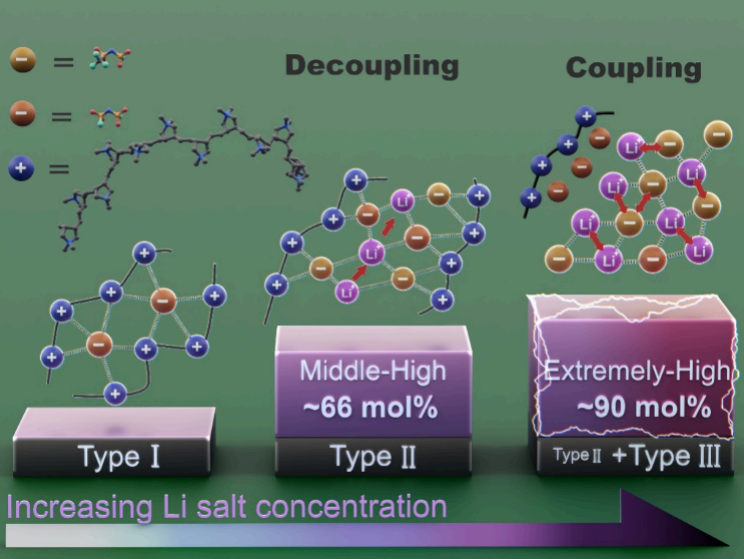

Polymer-in-salt electrolytes were introduced three decades ago as an innovative solution to the challenge of low Li-ion conductivity in solvent-free solid polymer electrolytes. Despite significant progress, the approach still faces considerable challenges, ranging from a fundamental understanding to the development of suitable polymers and salts. A critical issue is maintaining both the stability and high conductivity of molten salts within a polymer matrix, which has constrained their further exploration. This research offers a promising solution by integrating cationic poly(ionic liquids) (polyIL) with a crystallization-resistive salt consisting of asymmetric anions. A stable polymer-in-salt electrolyte with an exceptionally high Li-salt content of up to 90 mol % was achieved, providing a valuable opportunity for the in-depth understanding of these electrolytes at an extremely high salt concentration. This work explicates how increased salt concentration affects coordination structures, glass transitions, ionic conductivity, and the decoupling and coupling of ion transport from structural dynamics in a polymer electrolyte, ultimately enhancing electrolyte performance. These findings provide significant knowledge advancement in the field, guiding the future design of polymer-in-salt electrolytes.

## Introduction

Solvent-free solid polymer electrolytes (SPEs) are emerging as solid-state ionic conductors for electrochemical devices, offering enhanced safety and cycle performance in lithium (Li) batteries, by replacing volatile organic solvents with polymers.^1^ While this concept has been acknowledged for several decades,^2,3^ its widespread implementation remains hampered by the low cation conductivity. Traditional SPEs such as poly(ethylene oxide) (PEO) exemplify a coupled ion transport system, where the ion species, particularly Li ions, migrate through the polymer structural (segmental) dynamics. This coupled transport, due to strong ion-dipole interactions between the Li ion and polymer, results in a lower Li-ion transference number (<0.5) and brings significant internal polarization and premature cell failure for the corresponding Li batteries.^4^

Polymer-in-salt electrolytes (PISEs) with a high Li-salt concentration (>50 mol %) are a promising approach to overcome this challenge.^5−7^ Angell et al. first proposed the concept that a low-mole-percentage polymer can be added into a highly conducting salt solution to form a rubbery and highly conductive glassy electrolyte.^8−11^ The salt is the dominant component in this system, enabling the partial decoupling of ion conduction from polymer dynamics. However, the Li salts and polymer initially chosen by Angell are not suitable for batteries. Subsequent research by him and others has been devoted to exploring other polymers and salts.

Numerous neutral polymer materials (such as polyacrylonitrile (PAN), poly(ethylene carbonate) (PEC), and poly(ethylene oxide) (PEO)) have been applied to PISEs, demonstrating both relatively high ionic conductivity (*>*10^–6^ S cm^–1^ at 30 °C) and Li-ion transference number (>0.5).^12−15^ In parallel, the combination of ionic liquids and polymers as PISEs was also demonstrated to realize the decoupled ionic motion.^16−19^ However, the correlation between the complex coordination structures and coupling–decoupling of ion transport and the effect of salt concentration in PISEs remains unclear. Recently, cationic poly(ionic liquid)s (polyIL) have been utilized in the field of PISEs, termed polyIL-in-salt electrolytes.^20−23^ In such systems, the strong ion–ion interaction between the anion and polycation does not directly restrict the Li-ion motion. Our previous work suggests that a dominant coordination structure among the metal ion, anion, and polycation formed at high salt concentrations can facilitate metal-ion transport in these electrolytes.^21^ Wang et al. demonstrated that the polydiallyldimethylammonium bis(fluorosulfonyl) imide (PDADMAFSI) system containing lithium bis(fluorosulfonyl) imide (LiFSI) exhibits an ionic conductivity of 7.0 × 10^–5^ S cm^–1^ and a Li-ion transference number of 0.56 at 80 °C with a moderately high salt content (i.e., [polycation]:[Li^+^] = 1:1.5, by mole).^20^ A higher Li-salt ratio exceeding 1:1.5 results in the formation of an anion–Li^+^ aggregation regime where some anions coordinate only with Li ions, potentially leading to further enhanced Li-ion diffusion, as predicted by molecular dynamics (MD) simulations. However, this regime, being thermodynamically metastable, undergoes crystallization within a few days, impeding experimental validation. Nevertheless, this work points us in a new direction to study the ion decoupling motion in polymer-in-salt systems, and it also motivates us to further push the boundaries of Li-salt content beyond previous thresholds and delve deeper into polyILs with stable molten-salt states.

**Figure 1:**
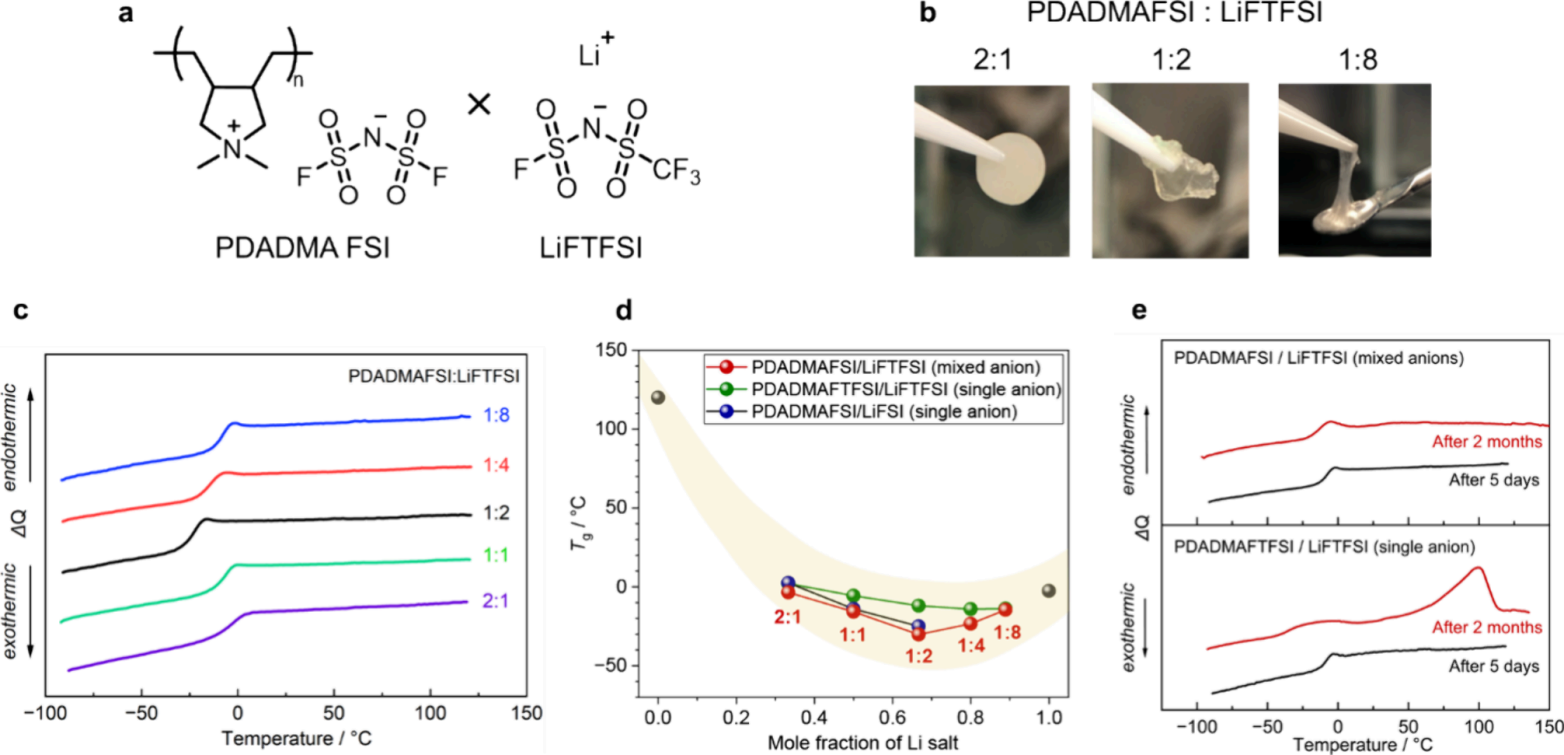
Thermal properties of the polyIL-in-salt systems. (a) Chemical structures of poly(diallyldimethylammonium) bis(fluorosulfonyl)imide (PDADMAFSI) and lithium (fluorosulfonyl)(trifluoromethanesulfonyl)imide (LiFTFSI). (b) Photographs of polymer electrolytes at the polyIL unit/LiFTFSI cation molar ratios of 2:1, 1:2, and 1:8. (c) DSC thermograms for polymer electrolytes with different LiFTFSI concentrations. (d) Relationship between *T*_g_ and the mole fraction of Li salt in both mixed anion system and single anion systems with either a FSI (up to 1:2) or a FTFSI anion (up to 1:8). (e) DSC thermograms of mixed anions and single FTFSI anion systems at a molar ratio of 1:8 after 5 days and 2 months.

In this study, by incorporating a glass-forming salt, lithium (fluorosulfonyl) (trifluoromethanesulfonyl) imide (LiFTFSI), into PDADMAFSI (Figure 1a), we successfully created a stable room temperature PISE with an extremely high salt concentration (i.e., [polycation]: [Li^+^] = 1:8, by mole; 90 mol % of Li salt). This offers an opportunity to investigate a polyIL-in-salt electrolyte in an extreme salt concentration range, as well as the effect of the use of mixed anions in this case. LiFTFSI is shown to be a good ionic conductor (>10^–4^ S cm^–1^ at 100 °C^24,25^) with a crystallization-resistant feature, helping maintain a glassy state in this polyIL-in-salt system. As the salt concentration increases, ion motion shifts from being decoupled (at a 1:2 ratio) to coupled (at a 1:8 ratio) with the structural dynamics, therefore affecting the glass transition temperature (*T*_g_). Promisingly, the overall ionic conductivity remains high at such a superhigh salt concentration, with an exceptionally high Li-ion transference number of 0.8. Furthermore, we show that the conductivity can be further optimized by changing the ratio of two anions while maintaining 90 mol % Li, due to the reduced *T*_g_, further improving ionic conductivity to 9.0 × 10^–5^ S cm^–1^ and Li transference number to 0.81 at 80 °C. The extreme polyIL-in-salt system shows the benefit of enhancing the electrolyte performance, including high oxidation stability, remarkable stability in Li deposition/dissolution cycling at a current density of up to 0.5 mA cm^–2^, and highly reversible charge–discharge cycling in prototype solid-state Li cells. Our finding provides critical insight into the interplay between salt concentration, coordination environments, and ion transport mechanisms in polyIL-in-salt systems across an unexplored middle-high to extremely high salt concentration range, with demonstrated enhanced electrolyte performance. This new understanding fills an important knowledge gap in the polymer-in-salt field, contributing to the design of highly metal-ion-conductive solid-state polymer electrolytes.

## Results and Discussion

### Thermal Properties

The mixtures of PDADMAFSI and LiFTFSI can form self-standing membranes at the polyIL cation/Li-ion molar ratio of 2:1, while the mechanical strength diminishes upon the increase of the salt content, ultimately transforming into a rubbery state at a 1:8 ratio (Figure 1b), as expected for polymer-in-salt systems. It is noted that PDADMAFSI is a rigid polymer, with its glass transition temperature (*T*_g_) around 120 °C.^26^ LiFTFSI is a low crystallinity salt, with a melting point (*T*_m_) of 106 °C and a glass transition temperature (*T*_g_) of −8.0 °C, as seen in differential scanning calorimetry (DSC) thermograms (Figure S1).^27^ Cleary, the asymmetric FTFSI shows an advantage in suppressing crystallinity compared to FSI. Only *T*_g_ was observed for LiFTFSI salt after the second heating, whereas crystallization (*T*_c_) and *T*_m_ were observed for LiFSI salt even after the second cycles.

DSC analysis in Figure 1c shows that all PDADMAFSI/LiFTFSI mixtures can remain completely amorphous, with a single *T*_g_ value. The *T*_g_ decreases monotonically with increasing Li-salt ratios from 2:1 to 1:2, reaching its lowest value of −30.2 °C due to the plasticizing effect of Li salt, i.e., the incorporation of Li salt weakens polycation–anion interactions via Li-ion-mediated cocoordination (polycation–anion–Li^+^), enhancing polymer chain dynamics.^21^ In addition, the low crystallinity of LiFTFSI can allow for higher Li-salt incorporation without inducing crystallization. As Li-salt content exceeds 1:2, the *T*_g_ of the system starts to increase gradually to approach that of pure LiFTFSI at −8.0 °C. Remarkably, the superhigh Li-salt composition, with a PDADMAFSI/LiFTFSI mole ratio of 1:8 (*m*_Li_= 89 mol %), exhibits no crystalline phase and maintains its molten-salt state even after 2 months at 25 °C (Figure 1e and Figure S1c). This is in stark contrast to the single FTFSI anion system, signifying the role of mixed anions in further enhancing the mixing entropy and, thus, improving the stability of molten salts. The overall relationship between *T*_g_ and the mole fraction of Li salt with mixed anions exhibits parabolic behavior (Figure 1d). In contrast, the single anion systems exhibit a more gradual reduction in *T*_g_, either with the FSI anion (up to 1:2) or with the FTFSI anion (up to 1:8), and in both cases, *T*_g_ is higher than that in the mixed anion system. The plasticizing effect on the polymer is insufficient to explain the parabolic behavior of *T*_g_ in the mixed anion system, making it essential to consider the ion–ion coordination structure at extreme salt ratios.

It should be noted that these polymer-in-salt electrolytes are considered to be in a thermodynamically metastable state. Therefore, sample preparation conditions and measurement intervals in subsequent experiments were kept consistent to ensure the reproducibility of each result.

### Ion Coordination Environment with MD Simulations

**Figure 2:**
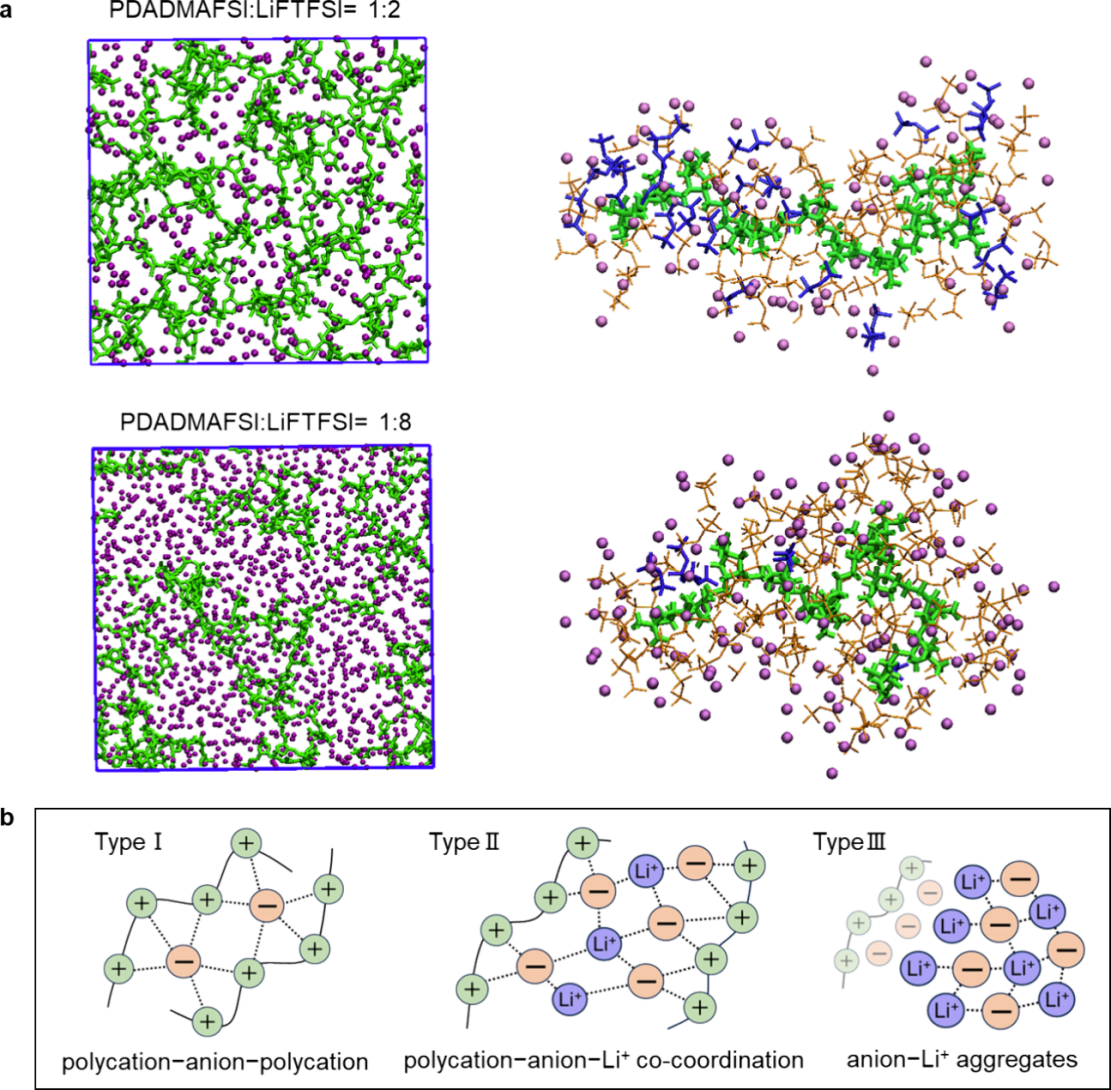
(a) MD snapshots showing the structures of two polyIL-in-salt systems at two salt concentrations and the coordination environment of a selected polymer segment at 1:2 and 1:8 systems. The simulation box snapshots show the distribution of Li ions (purple balls) and PDADMA chains (green sticks). The polycation chain segment snapshots show the surrounded cocoordinated anions and Li. FSI is in yellow bold sticks; FTFSI is in blue sticks. (b) Schematic diagram showing the three distinct anion coordination states with increasing salt concentrations.

We analyzed the ion coordination changes in PDADMAFSI/LiFTFSI electrolytes at 1:2 and 1:8 ratios using molecular dynamics (MD) simulation at 80 °C. Figure 2a shows the distribution of Li ions in the polymer matrix. At a 1:2 ratio, PDADMA chains and Li ions are homogeneously distributed throughout the simulation box, whereas at a 1:8 ratio, the lower polymer proportion suggests that salt-rich domains predominantly govern the structure. Three distinct anion coordination states in Figure 2b, denoted in previous research as Type I (polycation–anion–polycation), Type II (polycation–anion–Li^+^ cocoordination), and Type III (anion–Li^+^ aggregates),^20,21,28^ are quantified to analyze the ion–ion coordination environment (see Supplementary Note 1). Only Type II and Type III were identified in 1:2 and 1:8 systems, as Type I is normally associated with the neat polyIL or occurs at low salt concentrations. At a 1:2 ratio, around 95% of anions are Type II, nearing the maximum anionic cocoordination state, and only 5% are Type III. Increasing Li-salt content increases Type III coordination. At a 1:8 ratio, Type II decreases to 59.4%, and Type III increases to 40.6%, signifying a prominent anion–Li^+^ aggregation within the system. In this case, the *T*_g_ of the system begins to be influenced by the growth of the anion–Li^+^-aggregated component, and it gradually increases and approaches that of pure LiFTFSI (Figure 1d).

The detailed mixed anion coordination environments, distinguishing between FSI and FTFSI anions, were analyzed from their coordination numbers (CNs). At a 1:2 ratio, the CNs for FSI and FTFSI with PDADMA are 2.25 and 6.35 (around 1:3), respectively (Figure S3c and Table S2). They are 2.96 for FTFSI and 1.84 for FSI with Li. The ratio of the two anions in the first solvation shell of the cation is not fixed at 1:2. Clearly, there is more FTFSI in the PDADMA’s solvation shell, whereas FSI shows an advantage in competing with FTFSI for Li coordination. The mixed FTFSI and FSI coordination mitigates crystallization and further lowers the *T*_g_ at a 1:2 ratio compared to the systems with either single FSI^20^ or FTFSI anions (Figure 1d). At a 1:8 ratio, CNs are 9.3 for FTFSI and 0.46 for FSI with PDADMA and 4.46 for FTFSI and 0.6 for FSI with Li (Figure S3a and Table S2), showing that the FTFSI becomes predominant in both cation’s first solvation shell, due to its high number. The change in the coordination environment is shown in the snapshots of a selected polycation segment and its coordination environment in Figure 2a.

RDF analysis between Li^+^ and the anions reveals the three distinct peaks between 2 and 6 Å.^29,30^ The nearest peak at ∼2.5 Å is assigned to coordination between Li^+^ and negatively charged nitrogen atoms of anions. Larger peaks at 3.9 and 4.5 Å correspond to bidentate and monodentate coordination with oxygen atoms, respectively (Figure S3a). Monodentate coordination increases due to multiple Li coordinating with each anion at both 1:2 and 1:8 ratios, akin to behavior in highly concentrated ionic liquid electrolytes.^31^ It has been reported that the FTFSI anion plays a crucial role in inhibiting crystallization in highly concentrated liquid electrolytes.^32,33^ Reber et al.^34^ explained supercooling behavior in water-in-salt electrolytes with the FTFSI anion in MD simulation, noting that the monodentate coordination restricts N–SO_2_F bond rotation due to the preferential coordination of the Li ion with its S=O site, while the rotation of the N–SO_2_CF_3_ bond is relatively unrestricted. This leads to a greater rotational mobility of the N–SO_2_CF_3_ bond, resulting in a lower likelihood of crystallization. Similar behavior could occur in the polyIL-in-salt system, which helps hinder crystallization. We observe that the monodentate LiFTFSI coordination is more prevalent through the SO_2_F side than through the SO_2_CF_3_ side, as indicated by the Li–S RDFs calculated with the two S sites in Figure S3b,f. Further density function theory (DFT) calculations of CHelpG charges show that the F on the SO_2_F side is twice as negative as that on the SO_2_CF_3_ side (Figure S2), suggesting more delocalized negative charges on the SO_2_CF_3_ side, causing its weaker interaction with Li compared to the SO_2_F side. The conformational energy of different monodentate-coordinated LiFTFSI ion pairs also indicates more stable coordination geometries through the SO_2_F side.

### Ion Transport Properties

**Figure 3:**
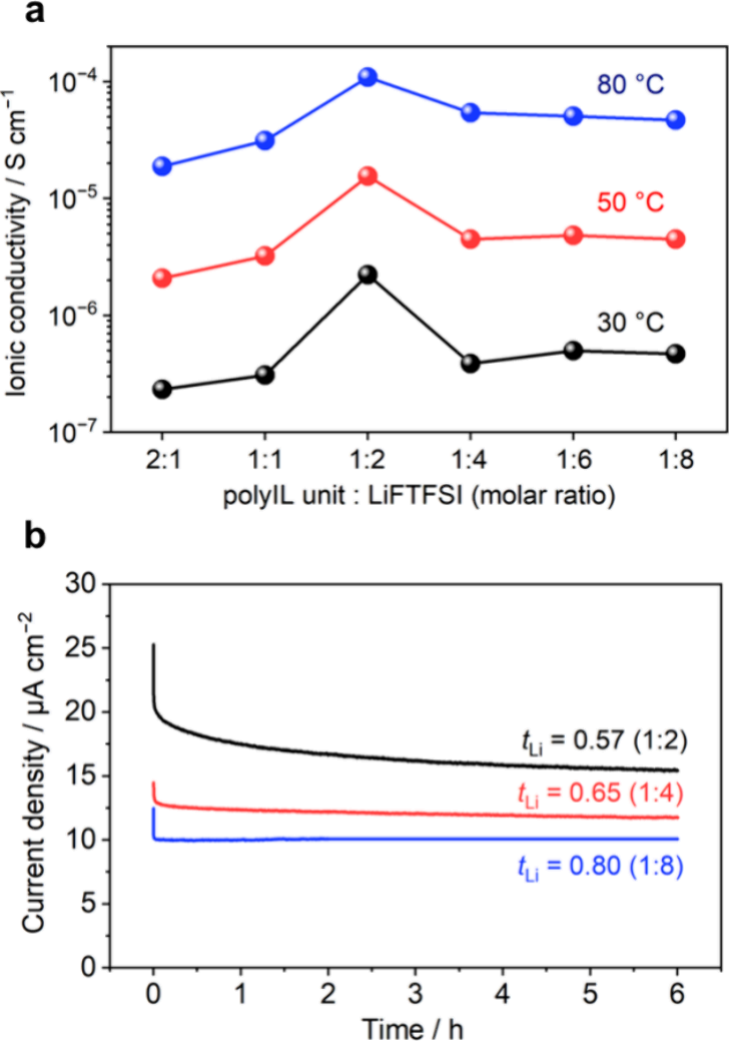
Ion transport properties of the polyIL-in-salt systems. (a) Ionic conductivities for different polyIL unit/LiFTFSI ratios at 30, 50, and 80 °C. (b) Current–time curve at 10 mV polarization and estimated Li-ion transference numbers for different polyIL unit/LiFTFSI ratios of 1:2, 1:4, and 1:8.

We explored the impact of elevated Li-salt concentrations on the ion transport for battery applications. Figure 3a presents the ionic conductivity of PDADMAFSI/LiFTFSI electrolytes at various polyIL unit/LiFTFSI cation molar ratios at 30, 50, and 80 °C. At 80 °C, ionic conductivity increases with Li-salt content from 2:1 to 1:2, peaking at 1.1 × 10^–4^ S cm^–1^ for the 1:2 ratio, which is higher than the highest conductivity obtained for this polyIL with the LiFSI salt (7.0 × 10^–5^ S cm^–1^). This increase corresponds to a decrease in *T*_g_ values (Figure 1d). Further increases in Li-salt content decrease the ionic conductivity to 4.7 × 10^–5^ S cm^–1^ at a 1:8 ratio, with a slight increase in *T*_g_. It is noteworthy that the decrease in the conductivity from 1:4 to 1:8 is minimal. At 30 and 50 °C, conductivity even slightly increases from 1:4 to 1:6, indicating that changes in ionic conductivity and *T*_g_ behavior are not fully aligned. This implies that a shift in dominant structural dynamics from polymer segmental dynamics to structural dynamics of the anion–Li^+^ aggregation domain affects the ionic conductivity, which will be discussed below.

Furthermore, the Li-ion transference number (*t*_Li+_), a crucial transport parameter for fast charge–discharge performance, was measured under anion blocking condition using a potentiostatic polarization method in a Li°||Li° symmetrical cell (see Supplementary Note 2).^4,35,36^Figure 3b shows the current–time curve at 10 mV polarization for different ratios, with impedance spectra and an equivalent circuit for estimating *t*_Li+_ in Figure S4. At a 1:2 ratio, *t*_Li+_ is 0.57, which is comparable to polyIL-in-salt systems using LiFSI.^20^ As the Li-salt content increases, the polarization current decay diminishes, mitigating concentration gradient formation in the cell. Notably, *t*_Li+_ increases continuously from a 1:2 to 1:8 ratio, achieving a maximum of 0.8 at a 1:8 ratio. Therefore, the benefit of suppressing crystallization at high salt concentrations is not only to prevent the conductivity decrease associated with crystallization but also to enhance *t*_Li+_. This indicates superior ion transport properties compared to those reported in recent studies on polymer-in-salt electrolytes (Table S3 and Figure S5).

### Coupling/Decoupling Ion Transport from Structural Dynamics

**Figure 4:**
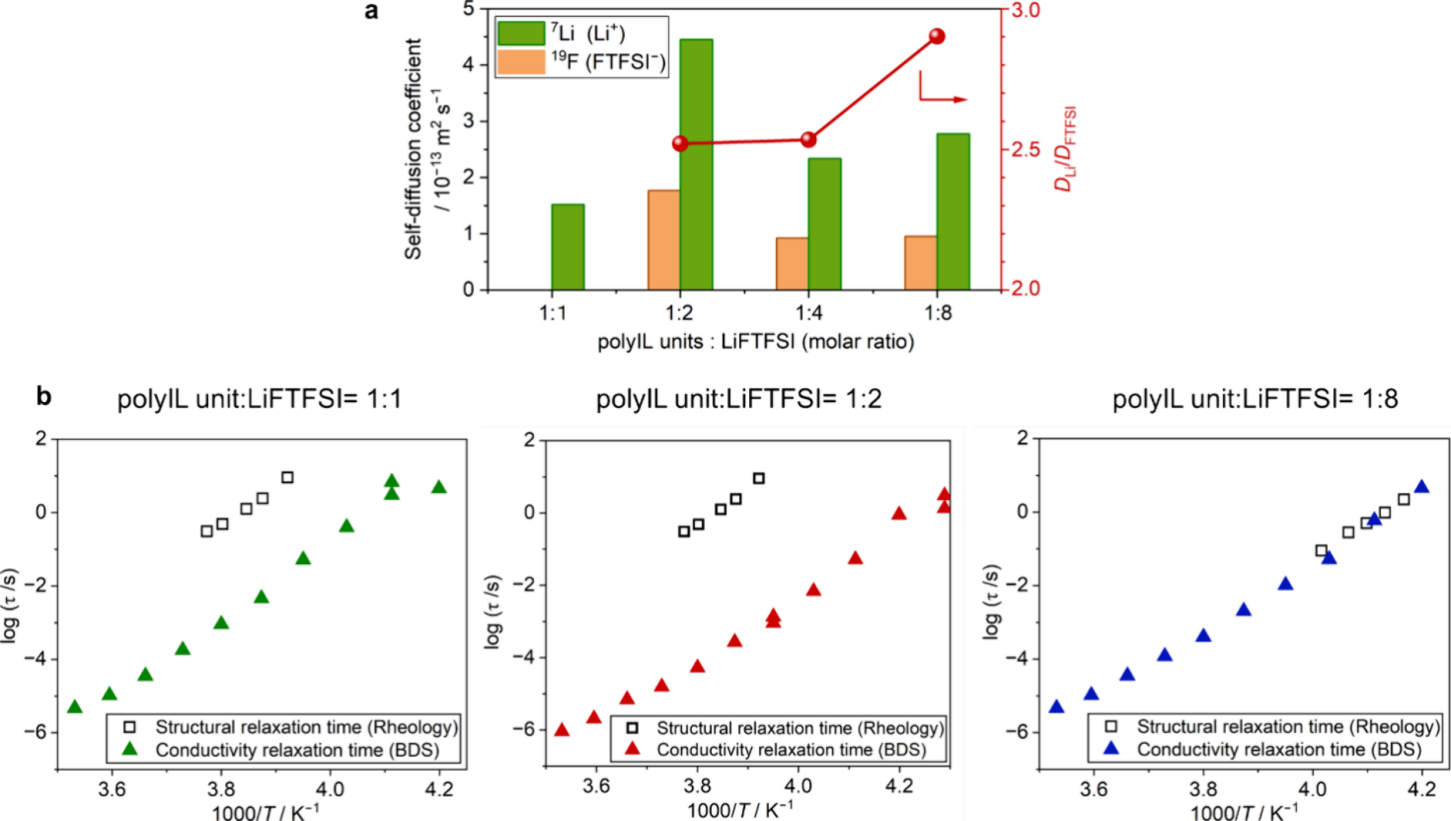
Analysis of ion transport behavior in the polyIL-in-salt systems. (a) Self-diffusion coefficients of different polyIL unit/LiFTFSI systems at 80 °C. (b) Temperature dependencies of structural relaxation time and conductivity relaxation time of different polyIL unit/LiFTFSI systems.

This section delves into the ion transport mechanisms within the polyIL-in-salt system, examining how to achieve a superior ionic conductivity. Pulsed-field gradient (PFG) NMR was used to measure the self-diffusion coefficients of Li^+^ ions (*D*_Li+_) and FTFSI anions (*D*_FTFSI_) at 80 °C as shown in Figure 4a. Unfortunately, the self-diffusion of the FSI anion could not be measured due to its short ^19^F transverse relaxation time (*T*_2_). *D*_Li_ and *D*_FTFSI_ increase as the Li-salt content shifts from a 1:1 to 1:2 ratio, with *D*_FTFSI_ at a 1:1 ratio being below the measurement limit. The increase in *D* is consistent with the increase in ion conductivity in Figure 3a. Increasing the Li salt to a 1:4 ratio lowers the diffusion, aligning with a decreased ionic conductivity and increased *T*_g_. Interestingly, from a 1:4 to 1:8 ratio, diffusion does not decrease, and the ratio of *D*_Li_ to *D*_FTFSI_ increases, despite an increase in *T*_g_ and a decrease in conductivity. This could suggest an increase in ion correlation at a 1:8 ratio, which will be discussed next.

MD simulations at 1:2 and 1:8 ratios and 80 °C provide further ion dynamics information (Figure S6). It is important to note that this analysis is not intended to accurately reproduce NMR diffusivity results, which are overestimated in this work, due to limitations in classical MD, including the use of a nonpolarizable force field, a simplified charge scaling method for polarization, and the significantly shorter polymer chains in the simulation compared to those in experiments. Nonetheless, this analysis still provides supportive and complementary information about relative ion diffusion measured by experiments. For example, mean square displacement (MSD) shows that Li^+^ ions move faster than other species at both ratios. As the ratio goes from 1:2 to 1:8, the displacement of all species decreases, but the relative motion ratio of the Li ion to the FTFSI anion increases, which supports the NMR results. This enhanced relative motion of Li ions, with an extremely high Li-salt concentration, boosts the Li-ion transference number (Figure 3b). Furthermore, MD simulations also suggest that the diffusion of FSI is higher than that of FTFSI, suggesting the possibility of enhancing ion conductivity by increasing the FSI component, which will be discussed in the next section.

We analyzed the conductivity relaxation time (τ_σ_) and the structural relaxation time (τ_s_) to further understand how ion transport couples or decouples from structural dynamics within the system across Li-salt concentrations. The temperature dependencies of τ_σ_ and τ_s_ were evaluated using broadband dielectric spectroscopy (Figure S7) and rheology (small amplitude oscillatory shear (SAOS) experiments, Figure S8), respectively. A detailed analysis is presented in Supplementary Note 3. These relaxation times follow a typical Vogel–Fulcher–Tamman (VFT)-type temperature dependence (Figure 4b), as observed in glass-forming materials.^37,38^ It is evident that τ_σ_ is consistently shorter than τ_s_ at 1:1 and 1:2 ratios in the measured temperature range, signifying a decoupling of ion transport from structural dynamics. Conversely, at a 1:8 ratio, τ_σ_ and τ_s_ are comparable, indicating a coupling of ion motion to structural dynamics. Based on these results, the structure at a 1:2 ratio facilitates the decoupling of Li-ion transport from structural relaxation essentially controlled by polymer segmental dynamics. In contrast, at a 1:8 ratio, the structural relaxation is dominated by the salt dynamics leading to the Li-ion transport coupling to the structural dynamics.

### Enhanced Electrochemical Performance via Mixed Anion Ratio Modulation

**Figure 5:**
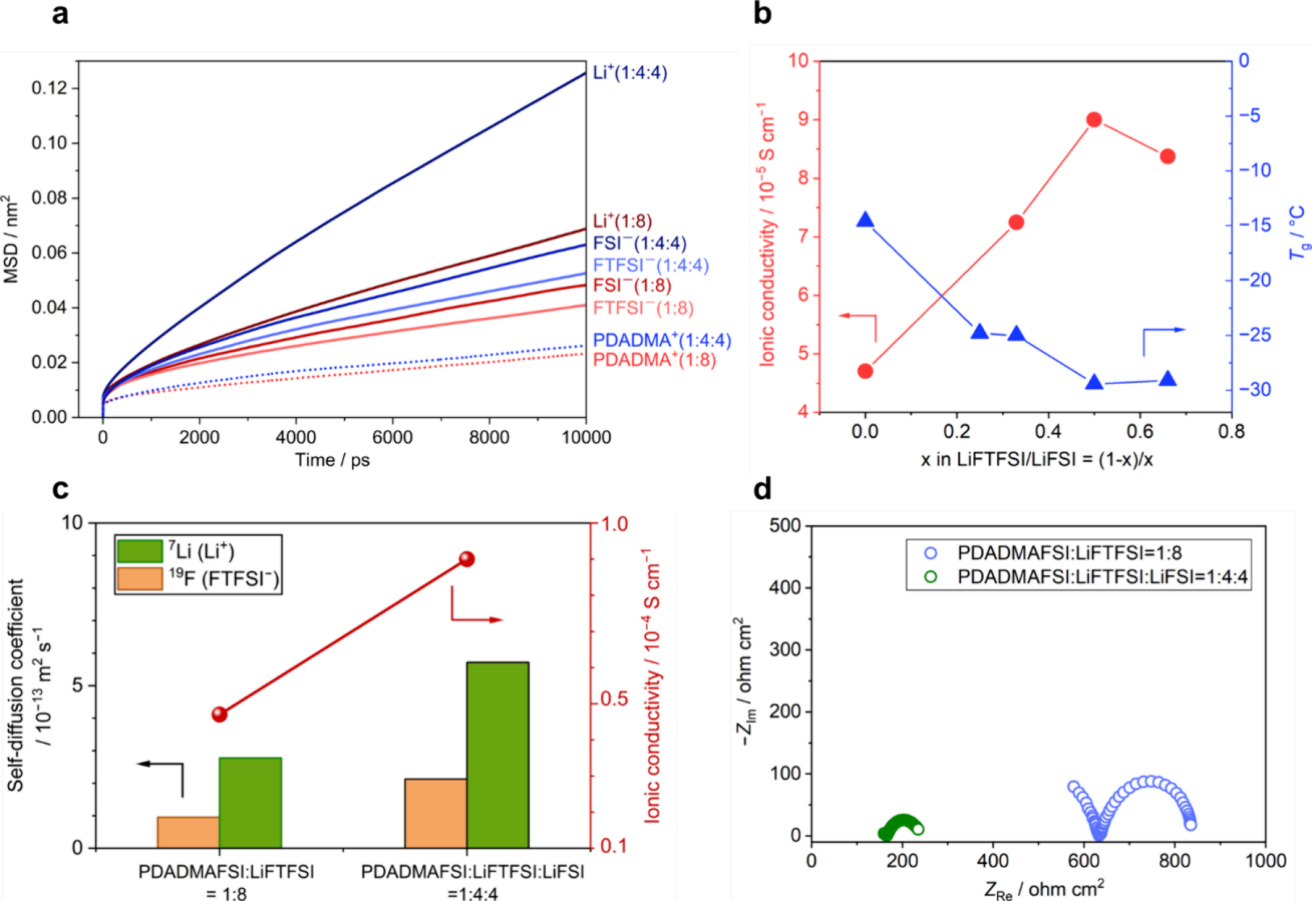
Mixed anion effect on ion transport and electrochemical properties. (a) MSD profiles obtained from MD simulations for Li^+^, FSI^–^, FTFSI^–^, and PDADMA^+^ at a PDADMAFSI/LiFTFSI ratio of 1:8 (red lines) and a PDADMAFSI/LiFTFSI/LiFSI ratio of 1:4:4 (blue lines) at 80 °C. (b) *T*_g_ and ionic conductivity measured as a function of the FSI mole fraction in mixed LiFTFSI/LiFSI systems with PDADMAFSI at polyIL unit:Li^+^ of a 1:8 ratio. (c) Experimental self-diffusion coefficients of FTFSI^–^ and Li^+^ and ionic conductivity at 80 °C. (d) Nyquist plots after the polarization for PDADMAFSI/LiFTFSI at a 1:8 ratio and for PDADMAFSI/LiFTFSI/LiFSI at a 1:4:4 ratio at 80 °C.

Since MD simulation suggests a faster FSI diffusion compared to FTFSI, it is reasonable to speculate that the Li^+^ diffusion could be enhanced through increasing the FSI component. To prove this, an MD simulation was conducted on a 1:4:4 PDADMAFSI/LiFSI/LiFTFSI system at 80 °C, which still maintains a high 1:8 salt concentration but changes the ratio of FSI to FTFSI from 1:8 to 5:4. MSD results in Figure 5a clearly show the enhancement of ion diffusion for all species in the 5FSI:4FTFSI compared to the 1FSI:8FTFSI. A systematic experimental study was followed by adjusting the FTFSI/FSI anion ratio while keeping the polyIL/Li^+^ ratio constant at 1:8. Figure 5b and Figure S9 illustrate the *T*_g_, ionic conductivity, and DSC thermograms of mixed LiFTFSI and LiFSI systems, combined with or without PDADMAFSI. The *T*_g_ of mixed Li salts without polycation correlates with that of the polyIL-in-salt system. In particular, the *T*_g_ of mixed salts gradually decreases as the LiFSI fraction increases, bottoming out at a LiFTFSI/LiFSI ratio of 1:1. An MD snapshot of the 1:4:4 system suggests that the FTFSI and FSI anions are coordinated with the polycation and Li in a mixed manner and are homogeneously distributed (Figure S10), mitigating crystallization and further lowering the *T*_g_. The experimental ionic conductivity of the polyIL-in-salt system increases as the *T*_g_ decreases, reaching a maximum of 9.0 × 10^–5^ S cm^–1^ at 80 °C (Figure 5b). This result is consistent with the increased diffusion coefficients of ionic species measured by MSD and PFG-NMR (Figure 5a,c). The *t*_Li_ remains high at 0.81 in the mixed system, which also exhibits significantly reduced interfacial resistance in the Li symmetrical cell (Figure 5d), likely due to a less resistive solid–electrolyte interface (SEI) originating from FSI decomposition.^39,40^

**Figure 6:**
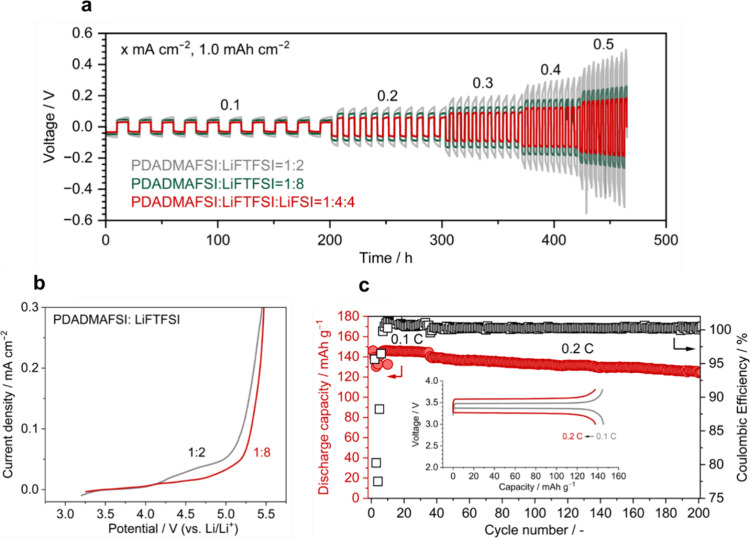
Electrochemical performance of the polyIL-in-salt systems at 80 °C. (a) Rate performance of Li deposition/dissolution cycling in the symmetrical Li cell with PDADMAFSI/LiFTFSI at ratios of 1:2 and 1:8, and PDADMAFSI/LiFTFSI/LiFSI at a 1:4:4 ratio. The enlarged view is presented in Figure S11. (b) Linear sweep voltammograms on a Pt electrode in PDADMAFSI/LiFTFSI at ratios of 1:2 and 1:8. (c) Discharge capacities and coulombic efficiencies of the Li/LiFePO_4_ cell using PDADMAFSI/LiFTFSI at a ratio of 1:8. Inset: charge–discharge curves of the cell.

To explore the impact of extremely high Li-salt concentrations in polyIL-in-salt systems on electrochemical properties for Li-metal batteries, we measured the galvanostatic cycling of a symmetrical Li°||Li° cell. This is a useful method to evaluate not only the electrochemical stability on the Li metal but also the bulk ion transport properties, unaffected by composite electrode fabrication. Cycling performance at current densities from 0.1 to 0.5 mA cm^–2^ for every 10 cycles at 80 °C, with a fixed Li deposition/dissolution of 1.0 mAh cm^–2^, revealed that PDADMAFSI/LiFTFSI at a 1:2 ratio exhibits an arcing behavior during Li deposition/dissolution at low current densities (Figure 6a). This behavior became more pronounced at a higher current density, which is attributed to the Li-ion mass transport limitation within the cell.^41−43^ At a 1:8 ratio, the slope of the voltage profile is more gradual with less polarization, although the conductivity decreases compared to the 1:2 system. This can be attributed to the extremely high Li-salt concentration and a high *t*_Li_ of 0.80 at a 1:8 ratio, mitigating the concentration gradient in the cell. The optimized mixed PDADMAFSI/LiFTFSI/LiFSI at a 1:4:4 ratio, with a higher conductivity and lower interfacial resistance, shows a lower polarization and stable Li deposition/dissolution profile up to 0.5 mA cm^–2^ without a short-circuit. The CV measurement shows enhanced oxidative stability up to ∼4.7 V with an increased Li-salt content (Figure 6b). This leads to highly reversible charge–discharge cycling in the LiFePO_4_ solid-state cell over 200 cycles with an average coulombic efficiency of >99.9% (Figure 6c). Thus, the overall improved electrolyte performance was demonstrated by pushing up the salt limit and using the mixed anions in a dry cationic polyIL/salt electrolyte.

### Outlook

Although this study has demonstrated the enhanced electrolyte performance by increasing the salt concentration limit, further improvement of the electrolyte performance for practical performance is still necessary. Here, we open a discussion on factors that should be considered for future design and optimization of polymer-in-salt electrolytes (PISEs). Using Li salts with low salt crystallinity is one key factor since high salt concentrations tend to form crystalline phases that reduce electrolyte conductivity. To suppress salt crystallization in polymer/salts melt electrolytes, increasing anion asymmetry is an effective way together with the use of mixed salts, which further lower the *T*_g_ of the electrolytes and enhance ionic conductivity.

Interestingly, in the polyIL-in-salt electrolyte, we found that the decoupled ion transport from structural relaxation in the medium-high salt concentration range (e.g., 1:2 here) changes to coupled ion transport at ultrahigh salt concentrations. Nevertheless, the electrolyte performance has improved. Therefore, it is not always necessary to pursue highly decoupled ion transport during electrolyte design. In addition, at extreme salt concentrations, the properties of Li salts become dominant factors in the overall electrolyte properties. We can see that both the *T*_g_ and conductivity approach those of the Li salts. Therefore, the excellent electrolyte properties of Li salts are crucial, such that the high conductivity of the Li salts should be the basic prerequisite.

On the other hand, the polymer should not significantly reduce the ionic conductivity of the PISEs. We believe that it is necessary to delve in-depth into the role of the polymer. According to our preliminary comparison of two ionic polymer matrices (polycation vs polyanion) at high salt concentrations, the nature of the polymer does show different effects on the physicochemical properties of electrolytes, and the polycationic electrolytes have obvious advantage in achieving high conductivity, which should be associated with interactions between polymers and salts. Future comprehensive studies and comparisons of various polymer electrolyte systems will certainly help provide more insights into the role of the polymer.

## Conclusions

In conclusion, we have developed poly(ionic liquid)s-in-salt systems at exceptionally high Li-salt concentrations by incorporating crystallization-resistant LiFTFSI into PDADMAFSI. A comprehensive investigation has been conducted, combining molecular dynamics simulations with experimental validation, including DSC, PFG-NMR, BDS, and rheology measurements. A specific focus was to clarifying the electrolyte behavior and different mechanisms involved in such a system. A notable transition from polycation–anion–Li cocoordination to an anion–Li^+^ aggregation-dominated coordination environment was unveiled as the polycations-to-Li ratio increases from 1:2 to 1:8. This transition significantly impacts the electrolyte *T*_g_, ion transport, and Li transfer number of the electrolytes. Our findings illustrate how the ion transport behavior shifts between decoupling and coupling with the structural dynamics. At a 1:2 ratio, a decoupling from structural dynamics dominated by the polymer is observed, transitioning to coupling with structural dynamics at a 1:8 ratio, due to the formation of anion–Li^+^ aggregates alongside the polycation–anion–Li^+^ cocoordination structure at increased salt concentrations. The Li-ion transference number significantly increases above 0.8. Nevertheless, ion motion is intricately affected by the structural dynamics and the *T*_g_. The *T*_g_ changes nonmonotonically with an increased salt concentration in the mixed anion system and can be further lowered at the highest salt concentration by modifying the ratio of two anions. This strategy facilitates structural motion and enhances ionic conductivity at the 1:8 salt system, thus maintaining a high Li-ion transference number. The presence of anion–Li^+^ aggregation domain was also shown to have a positive impact on improving battery performance compared to the previously reported 1:1.5 PDADMAFSI/LiFSI system, with enhanced Li conductivity, lowered interfacial resistance and polarization, and a long stable Li deposition/dissolution. Although further improvement in electrolyte performance is still required to achieve practical applications, an important contribution of this study is to demonstrate the feasibility of stabilizing molten salt by using asymmetric anions and mixed anions, which sheds light on strategies for designing a broad range of polymer-in-salt systems. More importantly, for the first time, ion coordination and coupling/decoupling transport in an unprecedented salt domain of cationic polymer electrolytes were clearly elucidated, greatly advancing knowledge in the field.

## Supporting Information Available

Experimental procedures, DSC thermograms, details of computational methods, DFT calculations, details of Li-ion transference number, MSD profiles, details of conductivity relaxation time and structural relaxation time, and ^1^H NMR spectrum (PDF)
