# Supplementary material for: Poly(Ionic Liquid) Electrolytes at an Extreme Salt Concentration for Solid-State Batteries
Source: J Am Chem Soc. 2024 Nov 19;146(48):33169–78. doi: 10.1021/jacs.4c12616 (PMC11636621; doi:10.1021/jacs.4c12616)
Supplement: Supplementary file 1 — ja4c12616_si_001.pdf [file ja4c12616_si_001.pdf]

Supplementary Information

Poly(ionic liquid) electrolytes at an extreme salt concentration for solid-state batteries

Shinji Kondou[1,2,3,4*], Mohanad Abdullah [5], Ivan Popov[6], Murillo L. Martins[5], Luke A. O'Dell[1,2], Hiroyuki Ueda[1,2], Faezeh Makhlooghiazad[1,2], Azusa Nakanishi[1], Taku Sudoh[4], Kazuhide Ueno[4,7], Masayoshi Watanabe[7], Patrick Howlett[1,2], Heng Zhang[8], Michel Armand[9], Alexei P. Sokolov[5,10], Maria Forsyth[1,2*], Fangfang Chen[1*]

[1] Institute for Frontier Materials, Deakin University, Burwood, VIC 3125, Australia

[2]ARC Industry Transformation Training Centre for Future Energy Technologies, Deakin University, Burwood, VIC 3125, Australia

[3] Department of Materials Engineering Science, Osaka University, 1-3, Machikaneyama, Toyonaka, Osaka 560-8531, Japan

[4] Department of Chemistry and Life Science, Yokohama National University, 79-5 Tokiwadai, Hodogaya-ku, Yokohama 240-8501, Japan

[5] Department of Chemistry, University of Tennessee, Knoxville, Tennessee 37996, United States

[6] University of Tennessee - Oak Ridge Innovation Institute, University of Tennessee, Knoxville, Tennessee 37996, United States

[7] Advanced Chemical Energy Research Centre (ACERC), Institute of Advanced Sciences, Yokohama National University, 79-5 Tokiwadai, Hodogaya-ku, Yokohama 240-8501, Japan

[8] Key Laboratory of Material Chemistry for Energy Conversion and Storage (Ministry of Education), School of Chemistry and Chemical Engineering, Huazhong University of Science and Technology Luoyu Road 1037, 430074, Wuhan, China

[9] Center for Cooperative Research on Alternative Energies (CIC energiGUNE), Basque Research and Technology Alliance (BRTA), 01510 Vitoria Gasteiz, Spain

[10] Chemical Sciences Division, Oak Ridge National Laboratory, Oak Ridge, Tennessee 37831, United States

Table of Contents

Experimental Procedures……………………………………..…………..……………………...……3

Figure S1………………………………………………………………………………..……..…… 7

Supplementary Note 1...……………...……………………………………..………………….............8

Figure S2 and Table S1…………………..……………………………….………………...………9

Figure S3 ……………...……………………..…..………………………………………...…… 10

Table S2..……………...…………………………….………………………………...…..….11

Supplementary Note 2……………………………………….……………………………..…..….12

Figure S4……………………………………………………………………….…………...…..13

Table S3 and Figure S5………………..…………………………………….………………...….....14

Figure S6……………………...……………………………………..………………………………15

Supplementary Note 3……………………………………….……………………………..…..….16

Figure S8………………...………….………………………………………………………………17

Figure S9………………...………….………………………………..………………………………18

Figure S10 and Table S3……………………..………………………………..……………...….….19

Figure S11……..………...…………….……………………………..……………………………20

Figure S12……..………...…………….……………………………..……………………………21

Experimental Procedures

Materials and Electrolytes Preparation. Lithium (fluorosulfonyl)(trifluoromethanesulfonyl)imide (LiFTFSI, 98%, Provisco CS, Czech Republic), lithium bis(fluorosulfonyl)imide (LiFSI, 99.9%, Solvionic), poly(diallyldimethylammonium) bis(fluorosulfonyl)imide (PDADMAFSI, 96%, M_n = 400 – 500 kDa, Polykey), poly(diallyldimethylammonium) chloride (PDADMACl, 20 wt% in H_2O, ca. M_n = 400 kDa, Sigma-Aldrich), potassium (fluorosulfonyl)(trifluoromethanesulfonyl)imide (KFTFSI, 98%, Provisco CS), acetonitrile (AN, 99.8%, Sigma-Aldrich) were used as purchased. Poly(diallyldimethylammonium) (fluorosulfonyl)(trifluoromethanesulfonyl)imide (PDADMAFTFSI) was synthesized through anion metathesis reactions using PDADMACl solution and KFTFSI.

The polyIL-in-salt samples were prepared by mixing polycation with Li-salts dissolved in AN at an appropriate ratio of an Ar-filled glove box. Subsequently, the solutions were subject to solvent evaporation and then vacuum dried for 48 hours at 50 °C, followed by 24 hours at 80 °C. The solvent residual of AN was carefully measured by ^{1}H NMR and was found to be completely removed at a ratio of 1:2. Even at a ratio of 1:8, it was only 0.029 wt% in the polymer electrolyte, which is considered to have no significant impact on the properties (Supplementary Figure 11). The mixed LiFTFSI/LiFSI salts were prepared by grinding them into a mortar, then melting them at 110 °C without solvent. All these procedures were carried out in an Ar-filled glove box.

Differential scanning calorimetry (DSC) measurement. Thermal properties of the polyIL electrolytes were measured using a Netzsch DSC (214 Polyma). 10–15 mg of samples was packed and

sealed in an Al pan in an Ar-filled glovebox. Cyclohexane was used for temperature calibration. All samples were measured at a heating rate of 10 °C min^{-1} in the temperature range of –100 to +150 °C.

Nuclear magnetic resonance (NMR) measurement. Self-diffusion coefficients of ionic species were studied using PFG NMR spectroscopy on a Bruker Avance III 300 MHz wide bore spectrometer equipped with Diff50 pulse field gradient probe at 80 °C. The samples were prepared inside the glovebox under Ar-filled atmosphere to prevent moisture contamination. The samples were loaded into a 4 mm NMR rotor for measurements.

Broadband dielectric spectroscopy (BDS) measurement. Conductivity relaxation time was evaluated by BDS in the frequency range of 10^{-2} to 10^{6} Hz, utilizing the Novocontrol system with an Alpha-A impedance analyzer and a Quatro Cryosystem temperature control unit. Samples were positioned between two gold-plated electrodes separated by a Teflon spacer. Before measurements, each sample was loaded on the bottom electrode and dried under vacuum at 50 °C for 2 days. The experimental process was carried out by gradually reducing the temperature from high to low levels. Initially, the samples were equilibrated at the highest temperature of 80 °C for at least 3 h, ensuring the reproducibility of the measurement. The samples were equilibrated at each temperature for 20 min before conducting the dielectric measurements.

Rheology measurement. Structural relaxation time was determined through small-amplitude oscillatory shear (SAOS) measurements in the linear regime, employing an AR2000ex rheometer (TA instruments). The experiments were conducted in the angular frequency range between 10^{-1} and

10^2 rad/s and temperature control was achieved using an environmental test chamber with nitrogen as the gas source. Before the experiments, the samples were dried at 50 °C for 2 days in a vacuum oven. Then they were placed in a 4 mm parallel plate geometry, heated up to 100 °C, and rapidly cooled down to temperatures below the respective T_g (~ −30 °C). Once at the lowest experimental temperatures, the samples were allowed to equilibrate for approximately 10 min to prevent temperature deviations larger than 0.2 °C and only then the experiments were performed.

Electrochemical measurement. Ionic conductivity was determined using an electrochemical impedance analyser (MTZ-35, Biologic) in the frequency range from 1 MHz to 100 mHz, with a voltage amplitude of 10 mV. Measurements were conducted using a sealed, in-house-designed dip cell that incorporated two platinum wires, and the cell constant was calibrated using a standard 0.01 M KCl aqueous solution at 25 °C. Li-ion transference number (t_{Li+}) was evaluated by the potentiostatic polarization method under anion-blocking conditions at 80 °C. [37, 45] The acetonitrile (AN)-based polyIL solution was impregnated into a glass fibre filter (∅ = 16mm, Whatman, Grade GF/C), followed by solvent evaporation under vacuum at 48 hours at 50 °C and 24 hours at 80 °C. A Li symmetrical cell was assembled using a glass fibre filter with polyIL electrolytes in a 2032 coin-type cell. The electrode of the Li metal was 1.54 cm^2 (∅ = 14mm). Electrochemical impedance measurements were performed in the frequency range from 1 MHz to 100 mHz, with a voltage amplitude of 10 mV at 80 °C, using a potentiostat (VMP, Biologic). Rate performance of Li metal deposition/dissolution cycling was measured using a symmetrical Li cell with an automatic charge

discharge instrument (Neware battery cycler) at 80 °C. The current densities ranged from 0.1 to 0.5 mA cm^{-2} every 10 cycles, and the amount of Li deposition/dissolution was fixed at 1.0 mAh cm^{-2}. The cathode composite electrode was prepared using $LiFePO_4$ powder (LFP; M121, Aleees) as the active material, carbon black (CB; Super C65, Imerys G&C) as the conduction supporting agent, 10wt% poly(vinylidenefluoride) (PVDF; Solef5130, Solvay) in N-methyl pyrrolidone (NMR; Sigma-Aldrich) solution as the binder, and 47 wt% PDADMAFSI/LiFTSFI (polyIL unit : LiFTFSI=1:8) in AN solution as the internal electrolyte. There materials were mixed in a mass ratio of LFP:CB:PVDF:PDADMAFSI/LiFTFSI=59:7:4:30 with an addition of NMP. The slurry from these materials was spread on an aluminium (Al) foil current collector and dried at 50 °C. The cell was assembled using the same method as in Li symmetrical cell. Galvanostatic charge−discharge measurements were performed at 80°C. The cut off voltages were set at 3.0 and 3.8 V. The specific capacity of the cell was calculated based on the active material loading (1.2 mg cm^{-2}-LFP). The oxidative electrochemical stability of the electrolytes was studied using linear sweep voltammetry (LSV) with a potentiostat (VMP, Biologic). LSV was performed at 80°C at a scan rate of 1 mV s^{-1} in a two-electrode cell with platinum (Pt) disk as the working electrode and Li foil as the counter & reference electrode.

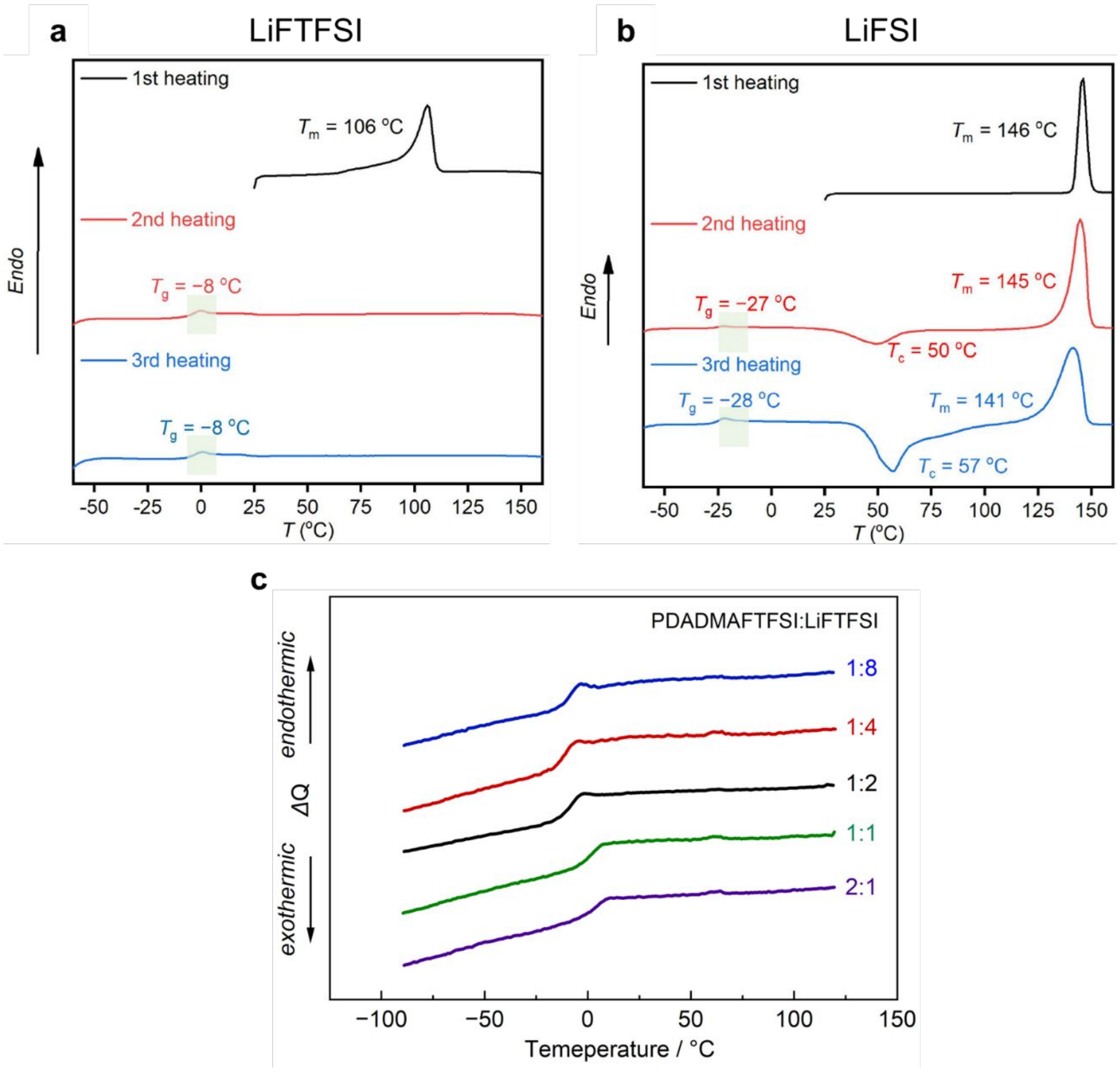

Figure S1. DSC thermograms: (a) pure LiFTFSI, (b) pure LiFSI, and (c) PDADMAFTFSI/LiFTFSI with different LiFTFSI content.

Supplementary Note 1: Computational Methods

MD simulations were conducted using Gromacs (Version 2022.22). The system consists of 15 Polycation chains with each having 12 repeat units. Three polyIL: salt concentrations were investigated including 1:2 and 1:8 of PolyDADMA FSI: LiFTFSI and 1:4:4 of PolyDADMA FSI: LiFSI: LiFTFSI. The initial simulation box was prepared using Packmol. The system was heated to 700 K first and equilibrated for 7 ns in an NPT ensemble. This enhances the velocity of ions and allows the system to fully relax and quickly reach the equilibrium, which will be checked through energy and volume profiles. Then the system was cooled down to 393 K and equilibrated for another 7 ns. During this annealing simulation, the temperature and pressure coupling methods are V-rescale and Berendsen. The tau-t and tau-p is set at 0.1 and 1.0, respectively. The pressure is 1 bar. Velocity integrator is chosen. All h-bonds are constrained. A 1 fs time step is used. Then the system was cooled down again to 353 K in an NPT ensemble and equilibrated for a total of 50 ns. For this simulation, the nose-hoover temperature thermostat and Parrinello-Rahman barostat methods were chosen to control temperature and pressure. The tau-t and tau-p is set at 1 and 2. The PME (Particle Mesh Ewald) method was used for handling electrostatic interactions with periodic boundary conditions. Fourierspacing is 0.12 nm and the pme-order is 4. The cutoff value of Coulombic interactions and van der Waals interactions is 1.2 nm. The 20 ns NVT simulation was followed for diffusion analysis.

The non-polarisable all-atom OPLS_AA force field [*JACS*, 1995, 117, 5179-5197] functional was chosen. The force field parameters of PDADMA were reported in our previous research (X. Wang *et al.*, *Joule*, 3, 2687–2702 (2019). & F. Chen *et al.*, *Nat. Mater.*, 21, 1175–1182 (2022)). The forcefield parameters of FSI and FTFSI are adopted from CL&P force field [*Theor Chem Acc* 2012, 131:1129], except for the atomic charges of FTFSI which were initially calculated using CHelpG charge scheme with Gaussian 09 software at the B3LYP/6-311++G(d,p) level of theory (**Figure S2)** . Then the charges were slightly modified to comply with the atomic charge on an equivalent site in TFSI anion in the CL&P force field. Table 1 listed all atomic charges for FSI and FTFSI in this research and those of TFSI are given as a comparison. A charge scaling factor of 0.7 was adopted to account for polarizable effect.

The anion-PDADMA and anion-Li coordination can be decided by comparing their distance with the coordination cutoff distance R1 or R2 marked in their radial distribution functions (**Figure S3**). This cutoff is usually the first prominent minimum value. The multiple minima for the first major coordination peak band are due to the different coordination geometries of anions with cations, for example, through N(FSI), bi-dentate (through O atoms on both sides of N) or monodentate (through O atoms on one side of N).

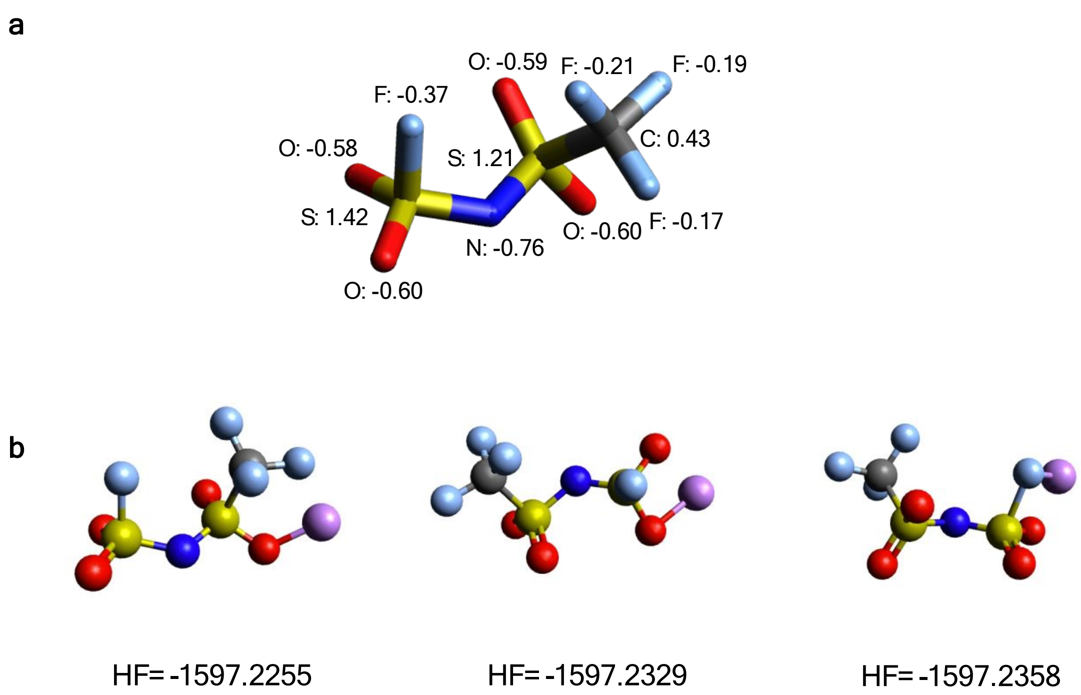

Figure S2. Density Functional Theory calculations at the B3LYP/6-311++G(d,p) level of theory. (a) CHelpG charges calculated for FTFSI; (b) Conformational energy of different LiFTFSI monodentate coordinated conformers (in Hartree).

Table S1. Atomic charges for FSI, TFSI and FTFSI used in the force field.

FSI	CL&P charge	TFSI	CL&P charge	FTFSI	Charge
N	-0.66	N	-0.66	N	-0.66
O	-0.53	O	-0.53	O	-0.53
S	1.02	S	1.02	S3 (with CF3)	1.02
F (-F)	-0.13	F (-CF3)	-0.16	F3(-CF3)	-0.16
		C	0.35	C3	0.35
				S2 (with F)	1.2
				F2(-F)	-0.31

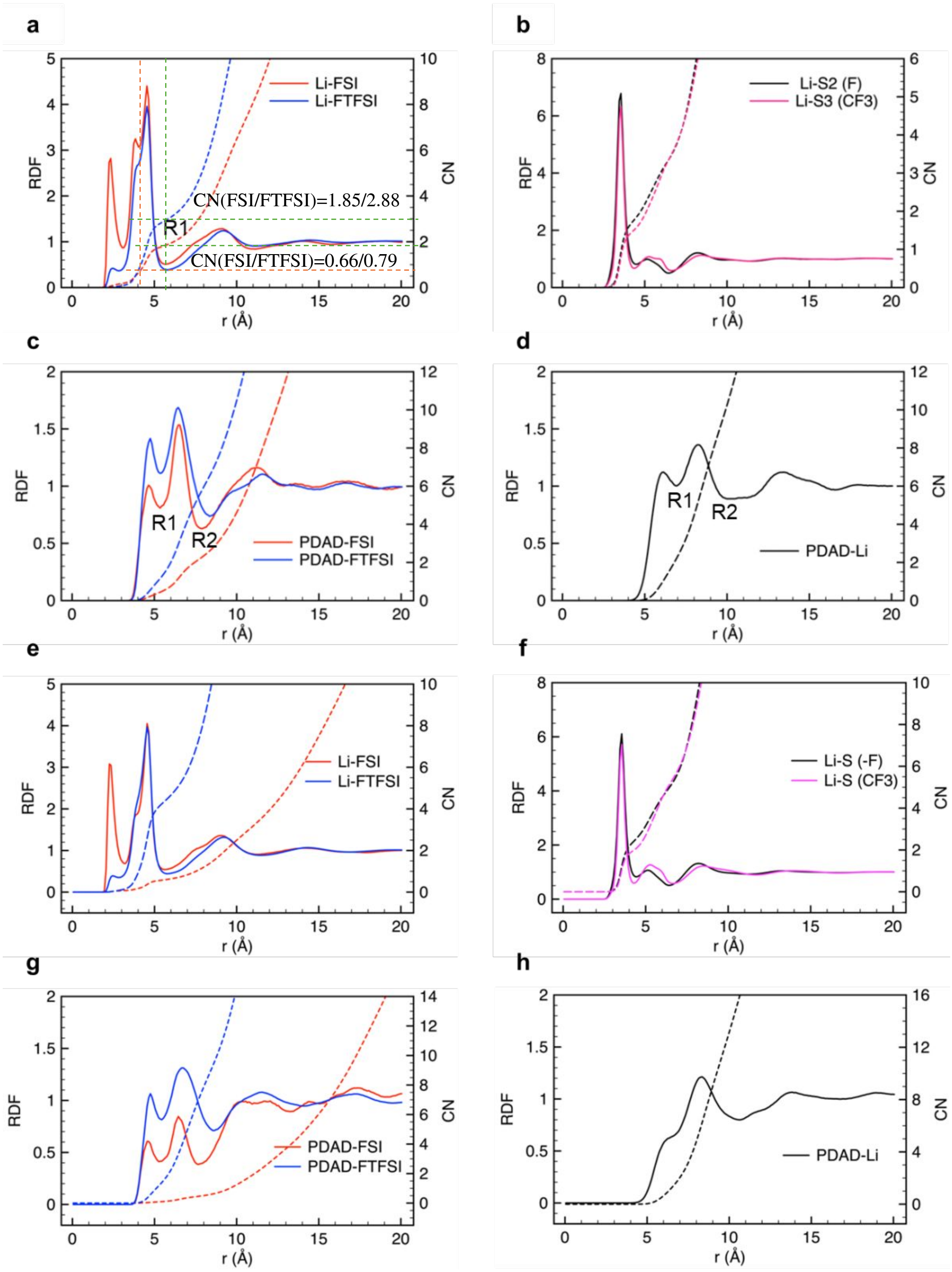

Figure S3. Radical Distribution Functions (RDF) and Coordination Number (CN) of Li-ions with N in FSI anions (red) and FTFSI anions (blue); Li ions with S2 (-F)(black) and S3 (-CF_3) (pink); nitrogen atom (N) in PDADMA chains with nitrogen atoms in FSI anions (red line) and FTFSI anions (blue line); N in PDADMA chains with Li-ions of (a-d) 1:2 and (e-h) 1:8 compositions, respectively. RDF and CN are plotted with solid and dotted lines.

Table S2. Coordination numbers at different cutoffs for the 1:2 /1:8 systems.

coordination	R1 (Å)	CN	R2 (Å)*	CN
Li-FSI	5.65 / 5.65	1.84 / 0.6		
Li-FTFSI	5.75 / 5.75	2.96 / 4.46		
Li-S2 (S-F)	4.45 / 4.45	1.82 / 2.55		
Li-S3(S-CF3)	4.35 / 4.25	1.51 / 0.09		
PDADMA-FSI	5.35 / 5.25	0.44 / 0.09	7.85 / 7.76	2.25 / 0.46
PDADMA-FTFSI	5.35 / 5.45	1.16 / 1.4	8.35 / 8.55	6.35 / 9.3
PDAD-Li	6.95 / 6.95	2.24 / 1.95	10.65	16.0

*The R1 and R2 distances of the polycation-anion coordination are due to different anon coordination geometries in polycation's first coordination shell.

Supplementary Note 2: Li-ion Transference Number ($t_{\mathrm{Li+}}$)

Equation 1 was then used to calculate the $t_{\mathrm{Li+}}$, according to the following equation: [1, 2]

$$t_{\mathrm{Li+}} = \frac{I_{\mathrm{SS}}(\Delta V - I_{\mathrm{ohm}} R_0)}{I_{\mathrm{ohm}}(\Delta V - I_{\mathrm{SS}} R_{\mathrm{SS}})} \quad (1)$$

where I_{ohm} and I_{SS} represent the initial and steady-state currents, respectively during polarization; ΔV denotes the constant applied voltage; and R_0 and R_{SS} are the initial and steady-state resistances, respectively, at the Li metal interface. In previous reports, $t_{\mathrm{Li+}}$ has been calculated using an initial current value, I_0, derived from the first data point on the polarisation current curve. However, Balsara[3] et al. reported that I_0 may not provide an accurate value in some cases due to its strong dependency on the data sampling time. Following this report, we adopted an alternative initial current (I_{ohm}) calculated by Ohm's law $I_{\mathrm{ohm}} = \Delta V/(R_{\mathrm{bulk}} + R_0)$, where R_{bulk} is the solution resistance for the calculation of $t_{\mathrm{Li+}}$. Although $t_{\mathrm{Li+}}$under anion blocking condition is considered to represent the true Li-ion transference number exclusively in ideal electrolytes, it can be more broadly defined as the 'current fraction' carried by Li ions under anion-blocking conditions and is a useful parameter to characterize the transport properties of electrolyte materials in LIBs.

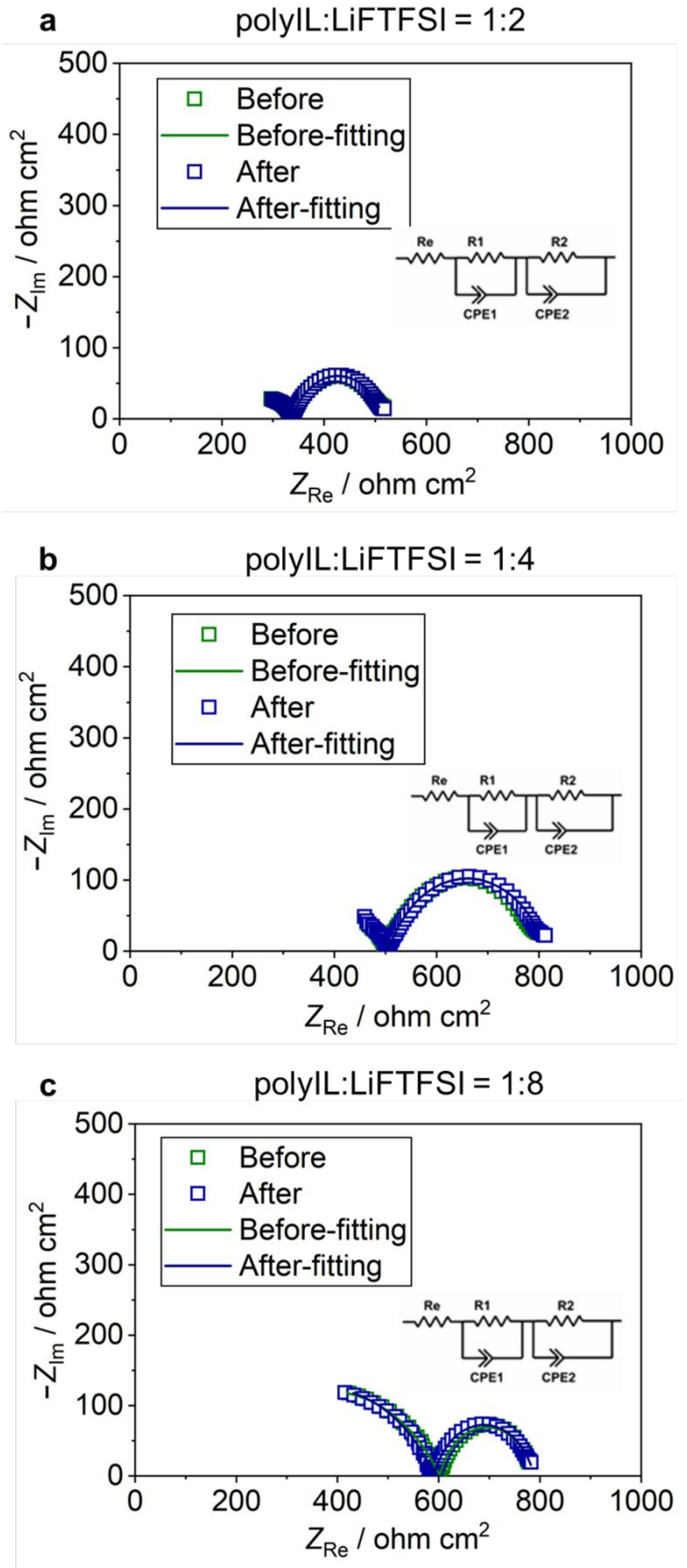

Figure S4. Nyquist plots before and after polarization of the symmetrical Li cell for different polyIL unit/LiFTFSI of (a) 1:2, (b) 1:4, and (c) 1:8 at 80 °C. Inset: The equivalent circuit used during fitting. The electrode area of the Li metal is 1.54 cm^2.

Table S3. Comparison of ionic conductivity and t_{Li} between this work and the previously published polymer-in-salt systems.

Electrolyte	Conductivity / S cm^{-1}	Li-ion transference number / -	References
LiTFSI : poly(3-(2-cyanoethoxymethyl)-3-ethyloxetane)=1:1	1.7×10^{-6}, 70 °C	0.7	[4]
LiFSI : poly(ethylene carbonate)=1.88:1	3.9×10^{-4}, 60 °C	0.54	[5]
LiTFSI/ poly(acrylonitrile-*co*-butyl acrylate)=1:1	1.0×10^{-4}, 85 °C	0.48	[6]
PDADMAFSI:LiFSI=1:1.5	7.9×10^{-5}, 60 °C	0.56	[7]
PDADMAFSI:LiFTFSI:LiFSI=1:2	1.1×10^{-4}, 80 °C	0.57	This work
PDADMAFSI:LiFTFSI:LiFSI=1:8	4.7×10^{-5}, 80 °C	0.80	This work
PDADMAFSI:LiFTFSI:LiFSI=1:4:4	9.0×10^{-5}, 80 °C	0.81	This work

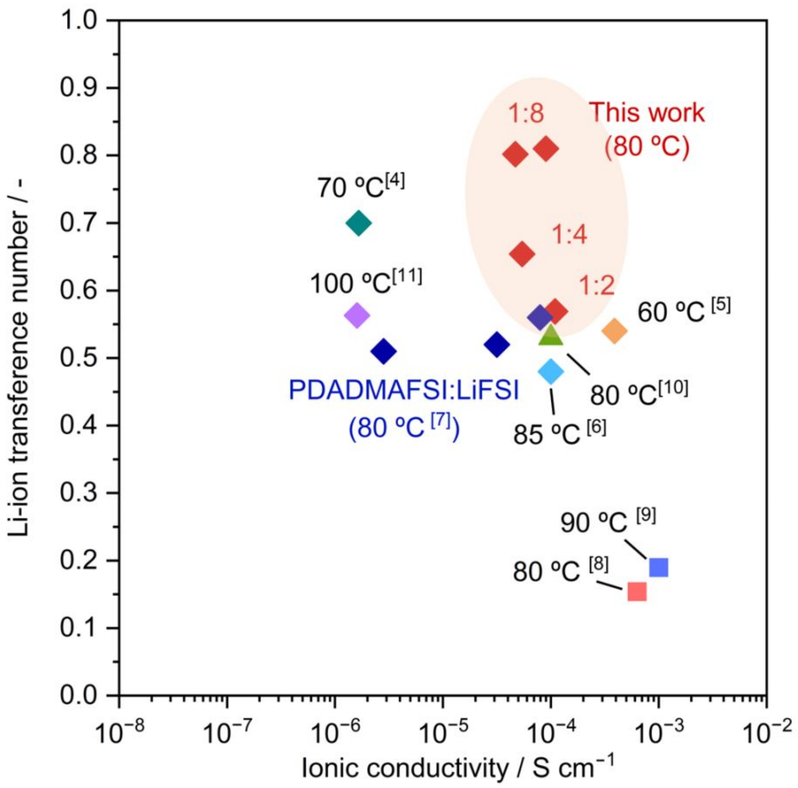

Figure S5. Relationship between Li-ion transference number and ionic conductivity for PDADMAFSI/LiFTFSI system in comparison to other polymer electrolytes (□:salt-in-polymer systems [8,9]; △ : weakly coordinating salt-in-polymer [10]; ◇:polymer-in-salt systems [4-7, 11]).

a polyIL unit : LiFTFSI=1:2

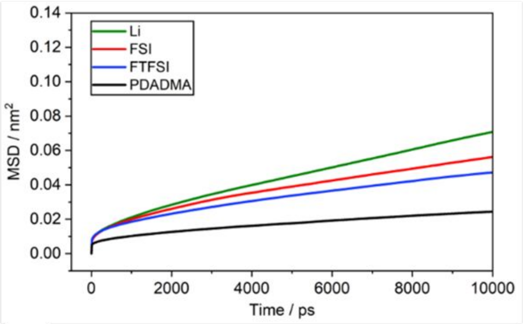

Species	Diffusion coefficient / $\times 10^{-13}$ m^2 s^{-1}	
	MD simulation	Experimental
Li^+	8.64	4.45
FSI^-	5.84	-
$FTFSI^-$	4.80	1.77
$PDADMA^+$	2.37	-

b polyIL unit : LiFTFSI=1:8

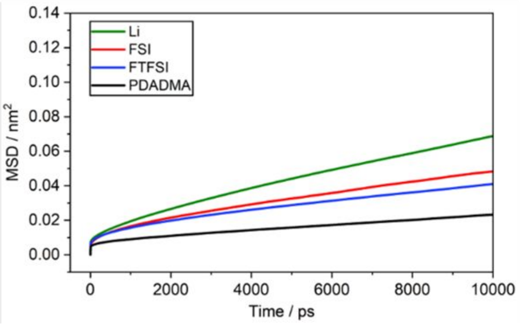

Species	Diffusion coefficient / $\times 10^{-13}$ m^2 s^{-1}	
	MD simulation	Experimental
Li^+	8.41	2.78
FSI^-	5.45	-
$FTFSI^-$	4.17	0.96
$PDADMA^+$	2.49	-

Figure S6. Mean square displacement (MSD) profiles for Li, FSI^-, $FTFSI^-$, and $PDADMA^+$ at polyIL/LiFTFSI molar ratios of (a) 1:2 and (b) 1:8 at 80 °C. The diffusion coefficients are obtained by fitting MSD profiles over a time range of 3000 to10000 ps. The tables compare diffusivities between MD simulations and experimental results.

Supplementary Note 3: Conductivity relaxation time and structural relaxation time

Typical conductivity spectra for polyIL unit/LiFTFSI systems are shown in Figure S6. The spectra demonstrate ordinary AC tails at high frequencies with DC plateaus at low frequencies (see Figure S6a). The AC-DC crossover is related to the transition of the ion sub-diffusion regime associated with ion rattling in the Coulombic cage to the normal Fickian diffusion regime when the ions overcome the highest potential barrier. The time scale of this crossover defines the conductivity relaxation and is characterized by a conductivity relaxation time, $\tau_\sigma=1/(2\pi f_{\text{AC-DC}})$, where $f_{\text{AC-DC}}$ defines the AC-DC crossover frequency. For a quantitative estimation of conductivity relaxation time, the Random Barrier Model [12] is applied according to the procedure shown in previous studies. [13]

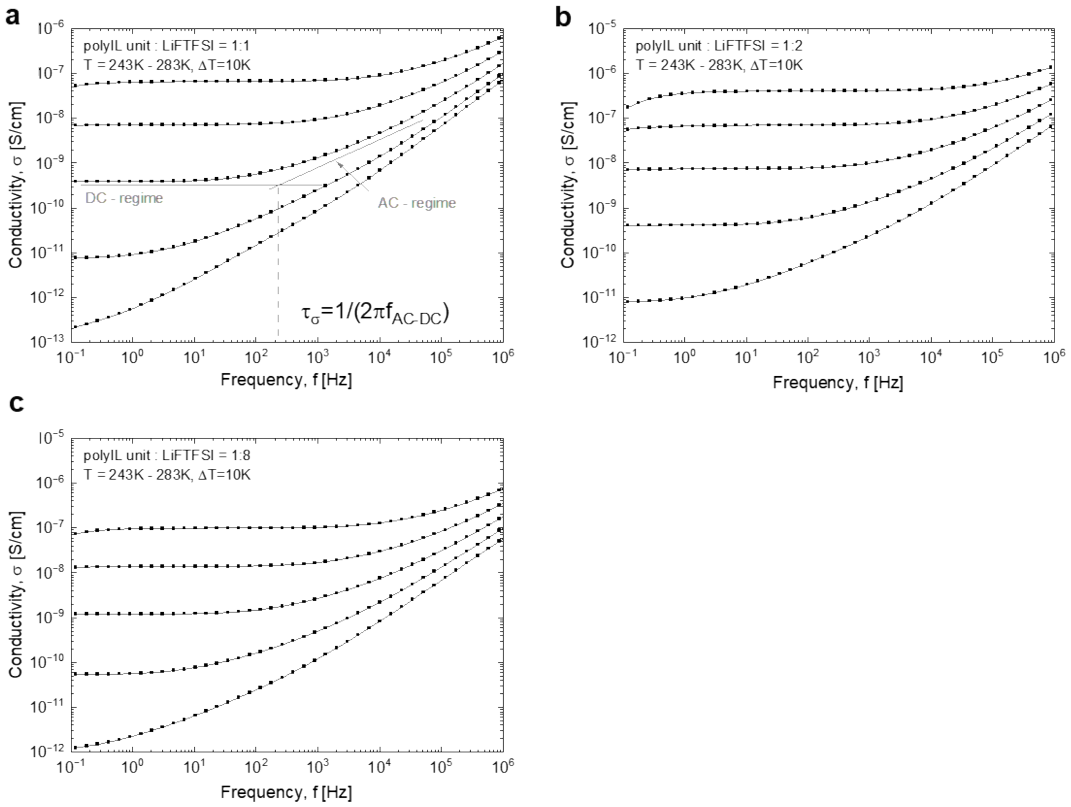

Figure S7. Conductivity spectra for polyIL unit/LiFTFSI systems at (a) 1:1, (b) 1:2, and (c) 1:8 ratios for selected temperatures.

The structural relaxation times were estimated from the SAOS experiments, using the crossover frequencies between G'(ω) and G"(ω) when the systems transition from the glassy to the viscoelastic regime. Note that we only considered the temperature range in which this process was within the frequency window of the instrument. Fig. 7 presents the master curves built for each mixture.

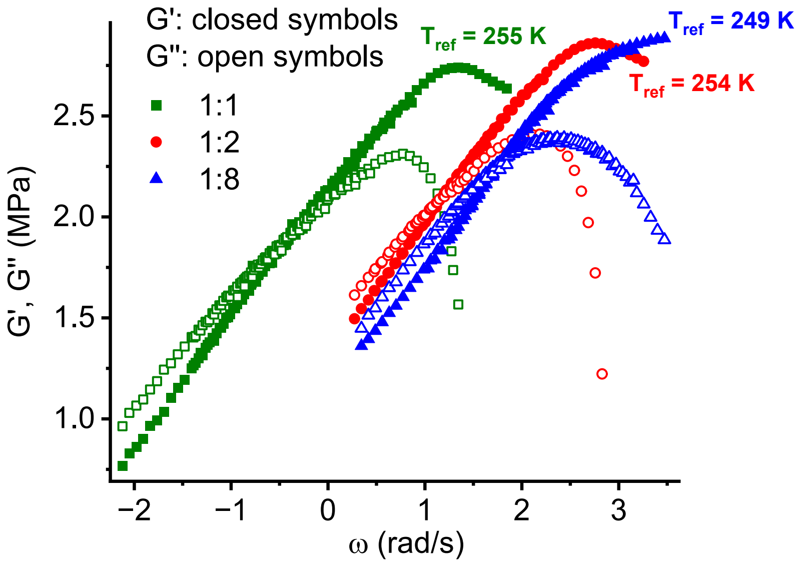

Figure S8. SAOS master curves of the different polyIL unit/LiFTFSI systems.

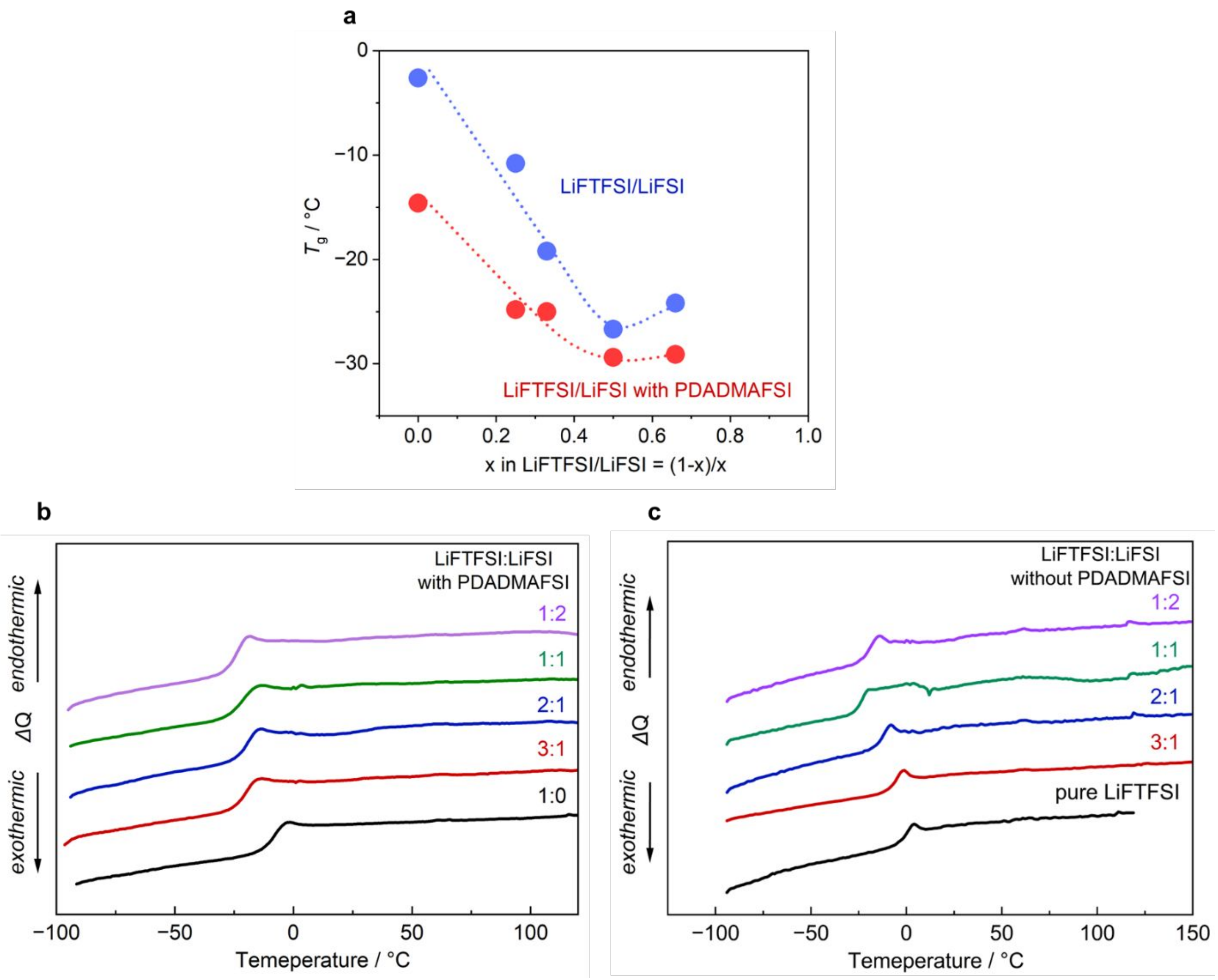

Figure S9. (a) Relationship between T_g and mole fraction of LiFSI in mixed LiFTFSI/LiFSI with and without PDADMAFSI. (b), (c) DSC thermograms of of mixed LiFTFSI and LiFSI systems at different ratios both with and without PDADMAFSI.

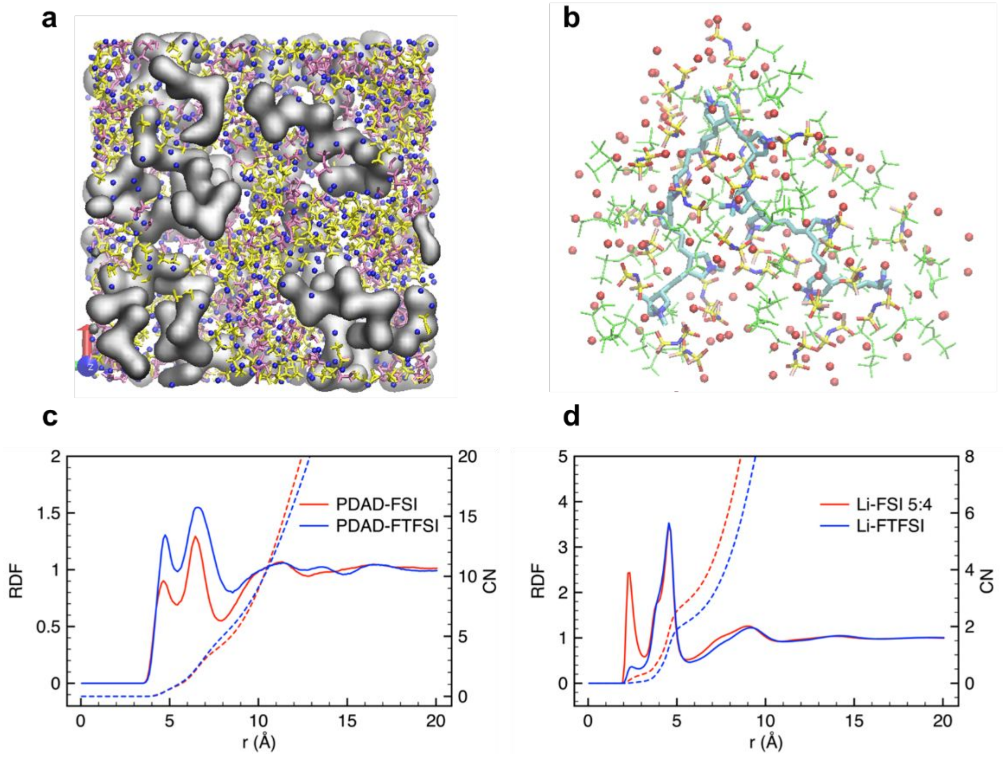

Figure S10. (a) Snapshot of the MD simulation box displaying the grey the polymer contour and Li[FSI][FTFSI] ion aggregates. The presented Li^+ and anions here are not coordinated with polycations. FSI: yellow; FTFSI:pink (b) Snapshot of a selected polycation chain and its coordination environment. FSI: yellow/blue/red, FTFSI: green; (c),(d) RDF and coordination number of nitrogen atom (N) in PDADMA chains with nitrogen atoms in FSI anions (red line) and FTFSI anions (blue line); Li-ions with N in FSI anions (red) and FTFSI anions (blue).

Table S4 Coordination numbers and cutoffs analysis for 1:4:4 system.

coordination	R1	CN	R2	CN
Li-FSI	5.55	2.87		
Li-FTFSI	5.75	2.23		
Li-S2 (S-F)	4.45	2.55		
Li-S3(S-CF3)	4.25	1.97		
PDAD-FSI	5.45	0.9	7.95	4.29
PDAD-FTFSI	5.45	0.97	8.55	5.89

PDAD-Li	6.85	2.76	10.05	15.4

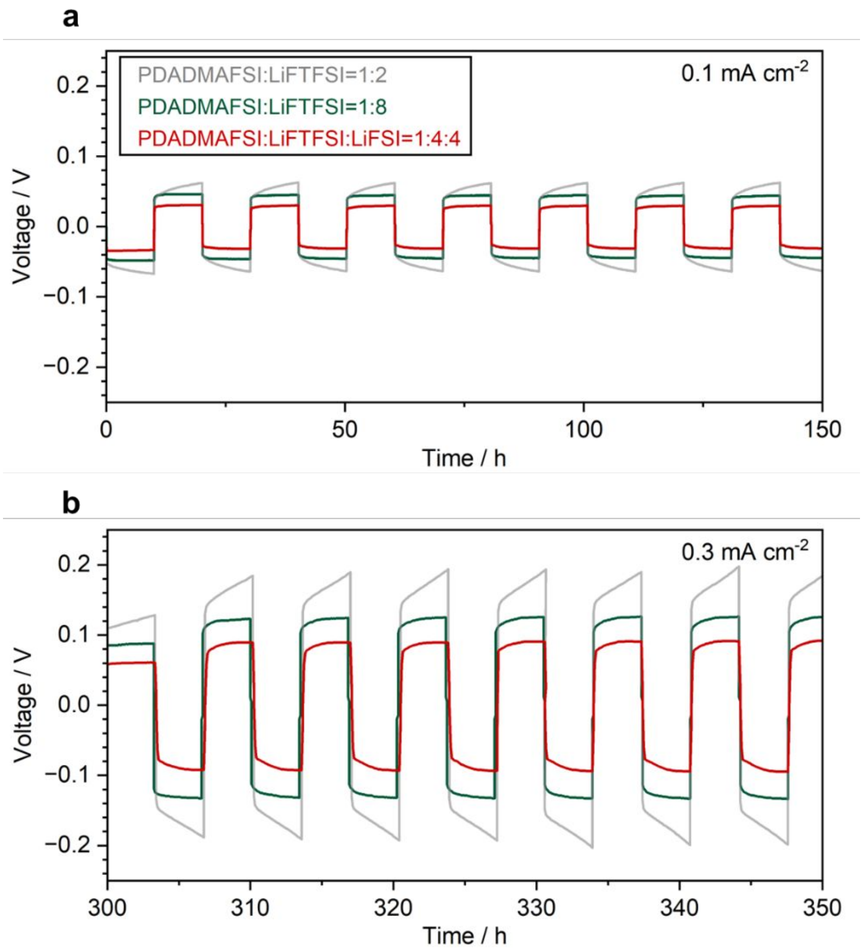

Figure S11. Enlarged view of Li deposition/dissolution cycling at (a) 0.1 mA cm^{-2} and (b) 0.3 mA cm^{-2} at 80 °C.

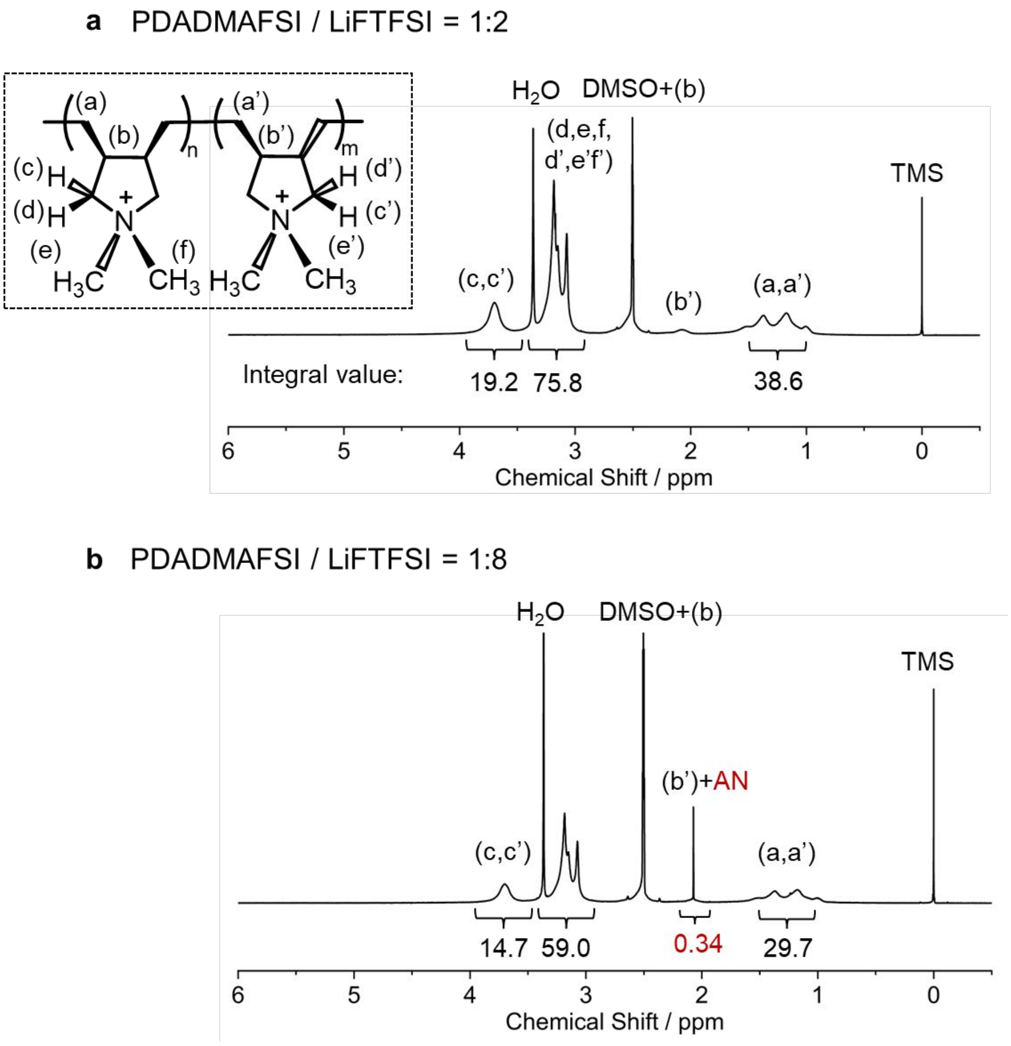

Figure S12. ^{1}H NMR spectrum and integral value of each peak of PDADMAFSI / LiFTFSI electrolytes at the polyIL unit / LiFTFSI cation molar ratios of (a) 1:2 and (b) 1:8 in DMSO-d6.

REFERENCES

1. Watanabe, M.; Nagano, S.; Sanui, K.; Ogata, N. Estimation of Li+ transport number in polymer electrolytes by the combination of complex impedance and potentiostatic polarization measurements. *Solid State Ionics* **1988**, *28-30*, 911-917.

2. Bruce, P. G.; Evans, J.; Vincent, C. A. Conductivity and transference number measurements on polymer electrolytes. *Solid State Ionics* **1988**, *28-30*, 918-922.

3. Galluzzo, M. D.; Maslyn, J. A.; Shah, D. B.; Balsara, N. P. Ohm's law for ion conduction in lithium and beyond-lithium battery electrolytes. *The Journal of Chemical Physics* **2019**, *151* (2), 020901.

4. Matsuoka, R.; Shibata, M.; Matsuo, K.; Sai, R.; Tsutsumi, H.; Fujii, K.; Katayama, Y. Importance of Lithium Coordination Structure to Lithium-Ion Transport in Polyether

Electrolytes with Cyanoethoxy Side Chains: An Experimental and Theoretical Approach. *Macromolecules* **2020**, *53* (21), 9480-9490.

5. Tominaga, Y. Ion-conductive polymer electrolytes based on poly (ethylene carbonate) and its derivatives. *Polymer Journal* **2017**, *49* (3), 291-299.
6. Florjańczyk, Z.; Zygadło-Monikowska, E.; Wieczorek, W.; Ryszawy, A.; Tomaszewska, A.; Fredman, K.; Golodnitsky, D.; Peled, E.; Scrosati, B. Polymer-in-Salt Electrolytes Based on Acrylonitrile/Butyl Acrylate Copolymers and Lithium Salts. *The Journal of Physical Chemistry B* **2004**, *108* (39), 14907-14914.
7. Wang, X.; Chen, F.; Girard, G. M.; Zhu, H.; MacFarlane, D. R.; Mecerreyes, D.; Armand, M.; Howlett, P. C.; Forsyth, M. Poly (ionic liquid) s-in-salt electrolytes with co-coordination-assisted lithium-ion transport for safe batteries. *Joule* **2019**, *3* (11), 2687-2702.
8. Hiller, M. M.; Joost, M.; Gores, H. J.; Passerini, S.; Wiemhöfer, H. D. The influence of interface polarization on the determination of lithium transference numbers of salt in polyethylene oxide electrolytes. *Electrochimica Acta* **2013**, *114*, 21-29.
9. Zheng, Q.; Pesko, D. M.; Savoie, B. M.; Timachova, K.; Hasan, A. L.; Smith, M. C.; Miller, T. F., III; Coates, G. W.; Balsara, N. P. Optimizing Ion Transport in Polyether-Based Electrolytes for Lithium Batteries. *Macromolecules* **2018**, *51* (8), 2847-2858.
10. Mackanic, D. G.; Michaels, W.; Lee, M.; Feng, D.; Lopez, J.; Qin, J.; Cui, Y.; Bao, Z. Crosslinked Poly(tetrahydrofuran) as a Loosely Coordinating Polymer Electrolyte. *Advanced Energy Materials* **2018**, *8* (25), 1800703.
11. Pożyczka, K.; Marzantowicz, M.; Dygas, J. R.; Krok, F. IONIC CONDUCTIVITY AND LITHIUM TRANSFERENCE NUMBER OF POLY(ETHYLENE OXIDE):LiTFSI SYSTEM. *Electrochimica Acta* **2017**, *227*, 127-135.
12. Dyre, J. C. The random free-energy barrier model for ac conduction in disordered solids. *Journal of Applied Physics* **1988**, *64* (5), 2456-2468.
13. Stacy, E. W.; Gainaru, C. P.; Gobet, M.; Wojnarowska, Z.; Bocharova, V.; Greenbaum, S. G.; Sokolov, A. P. Fundamental Limitations of Ionic Conductivity in Polymerized Ionic Liquids. *Macromolecules* **2018**, *51* (21), 8637-8645.
